# Hypochondroplasia gain-of-function mutation in FGFR3 causes defective bone mineralization in mice

**DOI:** 10.1172/jci.insight.168796

**Published:** 2023-06-22

**Authors:** Léa Loisay, Davide Komla-Ebri, Anne Morice, Yann Heuzé, Camille Viaut, Amélie de La Seiglière, Nabil Kaci, Danny Chan, Audrey Lamouroux, Geneviève Baujat, J.H. Duncan Bassett, Graham R. Williams, Laurence Legeai-Mallet

**Affiliations:** 1 Université de Paris Cité, Imagine Institute, Laboratory of Molecular and Physiopathological Bases of Osteochondrodysplasia, INSERM UMR1163, Paris, France.; 2 Molecular Endocrinology Laboratory, Department of Metabolism Digestion and Reproduction, Imperial College London, London, United Kingdom.; 3 UCB Pharma, Slough, United Kingdom.; 4 UMR5199 PACEA, CNRS, MC, Université de Bordeaux, Pessac, France.; 5 School of Biomedical Sciences, The University of Hong Kong, Pokfulam, Hong Kong, China.; 6 Department of Medical Genetics, CHU Arnaud De Villeneuve, Montpellier, France.; 7 Department of Medical Genetics, French Reference Center for Skeletal Dysplasia, AP-HP, Necker Enfants Malades Hospital, Paris, France.

**Keywords:** Bone Biology, Genetics, Bone development, Genetic diseases, Mouse models

## Abstract

Hypochondroplasia (HCH) is a mild dwarfism caused by missense mutations in fibroblast growth factor receptor 3 (FGFR3), with the majority of cases resulting from a heterozygous p.Asn540Lys gain-of-function mutation. Here, we report the generation and characterization of the first mouse model (*Fgfr3^Asn534Lys/+^*) of HCH to our knowledge. *Fgfr3^Asn534Lys/+^* mice exhibited progressive dwarfism and impairment of the synchondroses of the cranial base, resulting in defective formation of the foramen magnum. The appendicular and axial skeletons were both severely affected and we demonstrated an important role of FGFR3 in regulation of cortical and trabecular bone structure. Trabecular bone mineral density (BMD) of long bones and vertebral bodies was decreased, but cortical BMD increased with age in both tibiae and femurs. These results demonstrate that bones in *Fgfr3^Asn534Lys/+^* mice, due to FGFR3 activation, exhibit some characteristics of osteoporosis. The present findings emphasize the detrimental effect of gain-of-function mutations in the *Fgfr3* gene on long bone modeling during both developmental and aging processes, with potential implications for the management of elderly patients with hypochondroplasia and osteoporosis.

## Graphical abstract

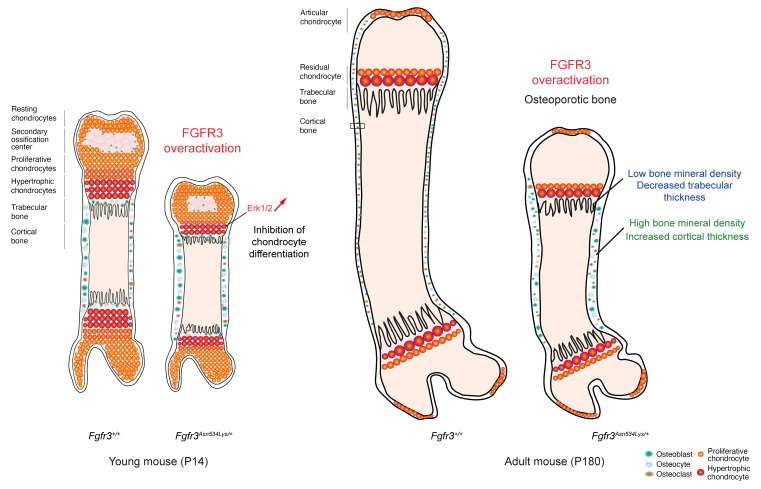

## Introduction

Hypochondroplasia (HCH, OMIM #146000) is an autosomal dominant skeletal disorder characterized by short-limbed dwarfism. The clinical and radiological features of this dwarfism are milder than achondroplasia (ACH, OMIM #100800), a severe form of dwarfism, and those of thanatophoric dysplasia (TDI, OMIM #187600; TDII, OMIM #187601), a lethal dwarfism. HCH is characterized by disproportionately short stature, rhizomelic short arms and legs, and mild joint laxity. Macrocephaly, prognathism, premature fusion of synchondroses, and foramen magnum stenosis are frequently reported in fibroblast growth factor receptor 3–related (FGFR3-related) osteochondrodysplasia (1–4). The radiologic features include shortening of the long bones with mild metaphyseal flare, narrowing of the inferior lumbar interpedicular distance, short broad femoral neck, and squared shortened ilia. Spinal defects are frequently reported in children and adults with ACH and HCH patients and are characterized by kyphosis, scoliosis, and lumbar lordosis due to anomalies of the vertebral bodies and intervertebral discs (IVDs) (5). Obstructive apnea and intellectual disability are also observed in HCH (6).

These clinical signs are generally less pronounced in HCH than those seen with ACH and may not be apparent until early or middle childhood. The incidence and prevalence of HCH is similar to that of ACH, which occurs in 1 in 15,000 newborns; more than 250,000 people worldwide have been diagnosed with HCH. Most HCH cases (70%) are caused by a common heterozygous gain-of-function mutation (p.Asn540Lys) in the tyrosine kinase domain of FGFR3 (7, 8). This receptor tyrosine kinase is a cell surface receptor that binds and responds to fibroblast growth factors (FGFs). These ligands bind to FGFR3, forming cross-linked dimers that trigger tyrosine kinase activation of FGFR3. FGFR3 gain-of-function mutations enhance the intracellular *trans*-autophophorylation of the activation-loop tyrosine kinase domain. Structural characterization studies highlighted the activating effect of the p.Asn540Lys mutation. This mutation drives the receptor into the active state by disengaging the autoinhibitory molecular brake at the kinase hinge domain (9–11). FGFR3 is a negative regulator of bone growth (12, 13) and missense *FGFR3* mutations induce defective long bone elongation due to abnormal proliferation and differentiation of growth plate chondrocytes. Many intracellular signaling pathways are triggered by FGFR3, among which are the mitogen-activated protein kinase (MAPK) pathway, which has been shown to regulate chondrocyte differentiation, and the signal transducer and activator of transcription protein (STAT) pathway that regulates chondrocyte proliferation (14). Their impact on chondrogenesis has also been shown in several *Fgfr3* mouse models recapitulating ACH, TDI, and TDII (15–21). FGF signaling controls not only chondrogenesis but also osteogenesis. FGFR1, FGFR2, and FGFR3 are expressed in both osteogenic lineages, periosteum and perichondrium (22), and in osteocytes isolated from cortical bone (23). Mice lacking *Fgfr1* and *Fgfr2* in mature osteoblasts or osteocytes display increased cortical bone remodeling and high bone mass (24), thus suggesting a critical homeostatic role for FGFRs in developing and adult bone. The role of FGFR3 in bone formation has been studied with gene inactivation in mice (*Fgfr3^–/–^*), which displayed decreased trabecular bone mass (25), and in a knockout zebrafish model *(fgfr3^lof/lof^*) that displayed a defect in the craniofacial skeleton formation (26). Mouse models of ACH with *Fgfr3* activation mutations (*Fgfr3^G369C/+^*, *Fgfr3^G380R/G380R^*, and *Fgfr3^Y367C/+^*) also exhibited defective bone formation, a decrease in femoral bone mass, and changes in bone microarchitecture in growing and adult mice (20, 27, 28).

It is well accepted that *Fgfr3* gain-of-function mutations play a key role in the malformation of the skeleton in both chondrodysplasia and craniosynostosis (29). Among the FGFR3-related osteochondrodysplasias, research programs have been developed using *Fgfr3* gene–targeted mouse models to provide mechanistic insights into ACH, with opportunities for therapy (14, 30). However, limited experimental and clinical studies have been conducted to determine the key features of the HCH phenotype, compounded by a lack of mouse models. So far, the lack of mouse models has restricted the progression in understanding the pathogenesis of HCH.

In this study, we generated the first mouse model of HCH to our knowledge, expressing a mutation homologous to that most frequently found in patients with HCH (p.Asn540Lys). This HCH mouse model (*Fgfr3^Asn534Lys/+^*) ubiquitously expresses the mutant N534K FGFR3 protein and exhibits mild dwarfism. Thus, the *Fgfr3^Asn534Lys/+^* mouse model offers a unique opportunity to study the impact of FGFR3 gain-of-function mutation in both endochondral and membranous ossification processes and to decipher its impact on skeletal development during childhood and adulthood. Here, we demonstrate that the presence of the *Fgfr3* p.Asn534Lys mutation impairs the function of both long bone cartilage and cranial synchondroses, resulting in defective bone growth and foramen magnum formation. Similarly, the spine was severely affected, with abnormalities of the IVD visible from birth and throughout adulthood. Unexpectedly, analysis of osteogenesis in adult *Fgfr3^Asn534Lys/+^* mice demonstrated opposing effects of the p.Asn534Lys mutation in the trabecular and cortical compartments of long bones. As observed previously in juvenile ACH and TD mice (20, 27, 28), the HCH gain-of-function mutation resulted in decreased trabecular bone mineral density (BMD) in tibiae, femurs, and vertebral bodies. However, in contrast, we observed increased tibial and femoral cortical BMD in adult *Fgfr3^Asn534Lys/+^* mice and demonstrated that long bones were brittle with reduced toughness. Consistent with this, transcriptional profiling of the femoral cortical bone demonstrated a downregulation of markers of the osteoblast lineage and high-resolution micro–computed tomographic (μCT) analyses of cortical bone highlighted anomalies of the osteocyte lacunae in the *Fgfr3^Asn534Lys/+^* mouse model of HCH. Altogether, these findings indicate that the N534K mutation leads to aberrant regulation of mature osteoblasts and osteocytes in trabecular and cortical bone.

Taken together, these data suggest that patients with HCH may have a previously unrecognized increased risk of fracture and this may have important implications for their medical follow-up and treatment during adulthood and aging.

## Results

### Fgfr3^Asn534Lys/+^ mice display a dwarf phenotype that recapitulates the clinical features of patients with HCH.

**Figure 1:**
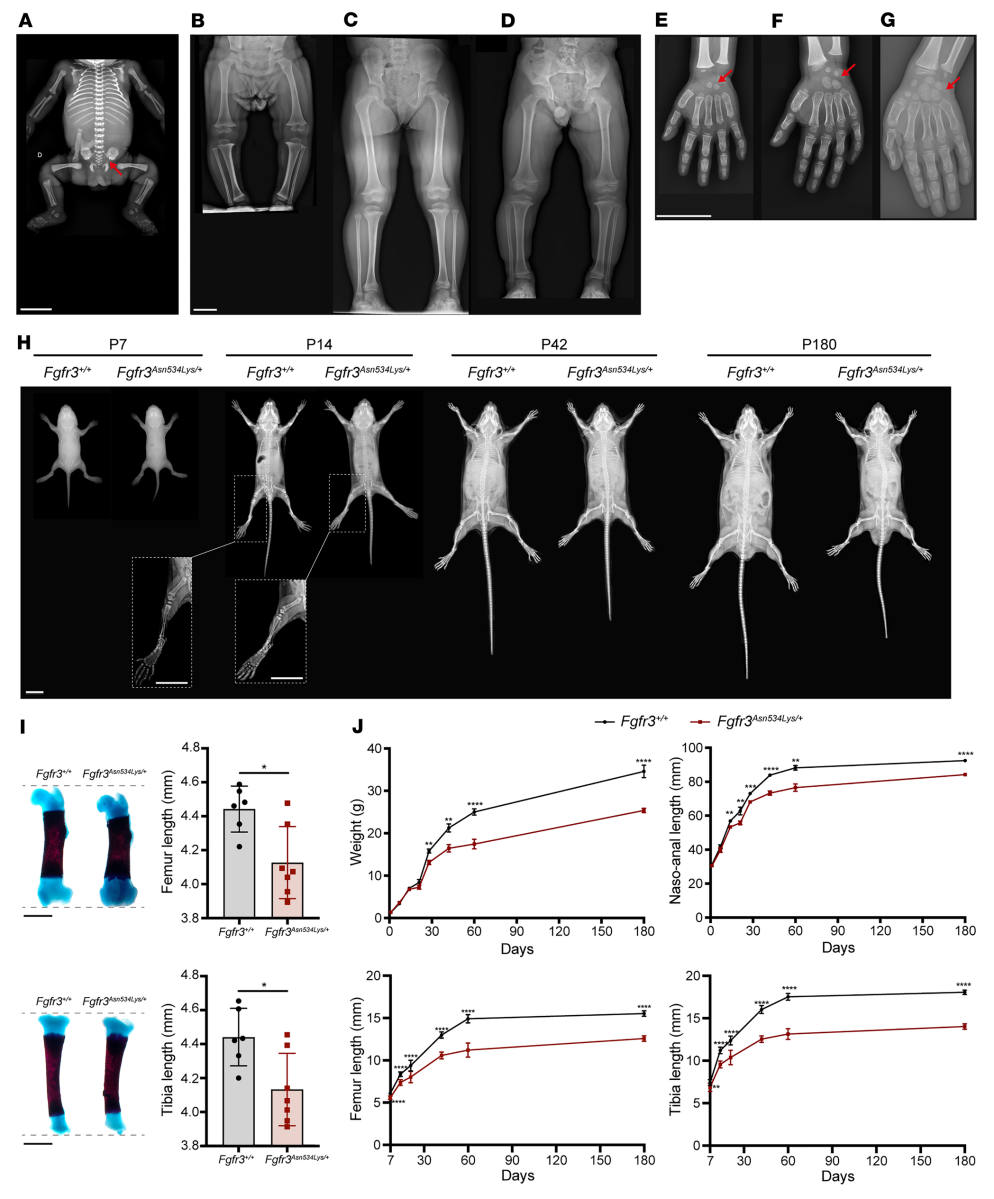
The hypochondroplasia mutation induces a dwarf phenotype in both human and mouse model. (**A**) X-rays of fetus (34 weeks of gestation) showing metaphyseal enlargement, trident acetabula, and squared ilia (red arrow) and a relative early cuboid ossification center appearance. (**B**–**D**) X-rays of HCH patients. (**B**) Three-year-old female showing slight tibial bending with elongation of the distal fibula. (**C**) Obliquity of the distal tibia metaphyseal growth plate of 5-year-old female. The leg bones are mildly short and the metaphyses are wide. (**D**) We noted an elongation of the distal fibula and broad internal ossified epiphyseal centers of the knee, squared iliac bones, and horizontal acetabular roofs in an 8-year-old male. Scale bar: 5 cm. (**E**–**G**) X-rays of patient hand extremities (**E**: 3-year-old female; **F**: 5-year-old male; **G**: 8-year-old male). We observed a bone carpal age delay (red arrows), a relative short and broad phalange (particularly the proximal and middle phalanges), and prominent ulnar styloid processes. Scale bar: 5 cm. (**H**) X-rays of both male and female *Fgfr3^Asn534Lys/+^* mice and their control littermates from 7 days to 180 days of age showing a progressive skeletal phenotype in *Fgfr3^Asn534Lys/+^* mice compared with *Fgfr3^+/+^*. Scale bar: 1 cm. (**I**) Alizarin red/Alcian blue staining of mouse femurs and tibiae on P1. Graphical representation of femur and tibia length. Scale bars: 1 mm. *Fgfr3^+/+^* (*n* = 6) and *Fgfr3^Asn534Lys/+^* (*n* = 7) P1 male and female mice. (**J**) Graphical representation of the body weight and naso-anal, femur, and tibia length of *Fgfr3^Asn534Lys/+^* and *Fgfr3^+/+^* male and female mice during development (P1, P7, P14, P21, P28, P42, P60, and P180). *P < 0.05, **P < 0.01, ***P < 0.001, ****P < 0.0001 by 2-tailed Student’s *t* test.

The skeletal phenotype of patients with HCH harboring the Asn540Lys mutation was investigated by x-ray (Figure 1, A–G). Shortened long bones with moderate rhizomelia (≤1 percentile) were visible by 34 weeks of gestation and metaphyseal enlargement, trident acetabula, and squared ilia were also identified (Figure 1A). The x-rays of 3-, 5- and 8-year-old HCH patients suggested significant variability in the clinical features of HCH (Figure 1, B–G). In the 3- and 5-year-old female patients (Figure 1, B and C) the height was –2 standard deviations (SD) below the age- and sex-matched mean and long bones were short and broad. By contrast, in the 8-year-old male patient (Figure 1D) the height was –4 SD below the age- and sex-matched mean and the fibula was disproportionately long, with obliquity of the distal tibia metaphyseal growth plate. The carpal bone age was also determined using the standard Greulich and Pyle atlas and was delayed in all patients. Furthermore, the ulnar styloid was yet not mineralized at this age, but we noted in the 3 patients a slight internal metaphysis enlargement of the distal ulna, with obliquity of the metaphyseal line, which leads to the styloid aspect frequently reported in older HCH patients (Figure 1, E–G).

To further understand the impact of the p.Asn540Lys mutation on the skeleton during growth, we generated a mouse model of HCH using Cre-*loxP* gene targeting. We introduced the most frequent HCH FGFR3 p.Asn534Lys mutation (corresponding to Asn540Lys in humans) in the *Fgfr3^Asn534Lys/+^* mouse model. Excision of the *loxP* cassette of the mouse line *Fgfr3^+/loxp^ ^Asn534Lys^* was achieved using the mouse line CMV-Cre (31) (Supplemental Figure 1A; supplemental material available online with this article; https://doi.org/10.1172/jci.insight.168796DS1). We confirmed the presence of the heterozygous Asn534Lys mutation in *Fgfr3^Asn534Lys/+^* mice by DNA sequencing (Supplemental Figure 1B). Detailed skeletal phenotyping of the *Fgfr3^Asn534Lys/+^* mice was performed at multiple time points from birth to adulthood. X-ray analysis demonstrated a progressive skeletal phenotype in *Fgfr3^Asn534Lys/+^* mice from postnatal day 7 (P7) until P180 (Figure 1H). Studying tibia and femur lengths at birth, we noted a reduced length in *Fgfr3^Asn534Lys/+^* compared with *Fgfr3^+/+^* mice (–6.93%, [*P* = 0.03] and –7.09% [*P* = 0.01], respectively) (Figure 1I). The length of long bones was reduced across all analyzed time points, with the largest difference recorded on P60 (–28.33% in femurs [*P* < 0.0001], –28.65% in tibiae [*P* < 0.0001]) (Figure 1J and Supplemental Table 1). Body weight and naso-anal length were also decreased in *Fgfr3^Asn534Lys/+^* mice, and no difference was seen between males and females within the genotype (Figure 1J, Table 1, and Supplemental Table 1). On P60, body weight was reduced by 35% in *Fgfr3^Asn534Lys/+^* compared with controls (*P* < 0.0001) (Figure 1J and Table 1). The phenotype severity was progressive, with the naso-anal length being similar to control on P7 (*P* = 0.451) but reduced by 12% on P21 (*P* = 0.0056), 14% on P60 (*P* < 0.0001), and 9% on P180 (*P* < 0.0001). These macroscopic and x-ray analyses demonstrate that the *Fgfr3^Asn534Lys/+^* mice recapitulate the phenotype of human HCH and exhibit a progressive dwarfism from birth and throughout adulthood.

### Fgfr3^Asn534Lys/+^ mice display characteristic craniofacial abnormalities.

**Figure 2:**
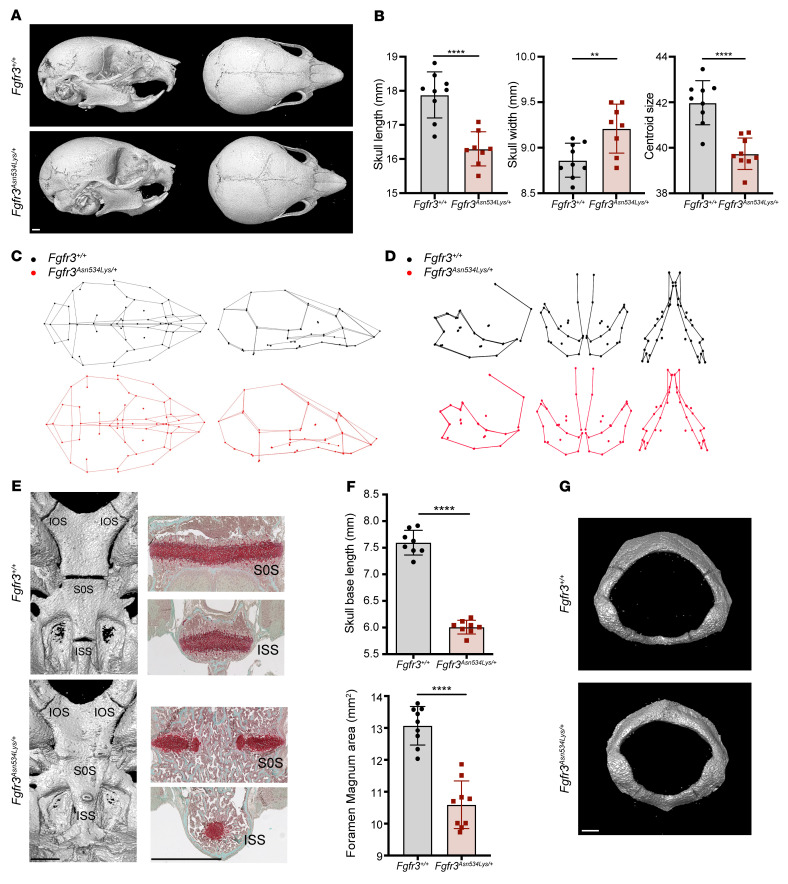
*Fgfr3^Asn534Lys/+^* mice exhibit craniofacial anomalies. (**A**) Representative *Fgfr3^+/+^* and *Fgfr3^Asn534Lys/+^* skull bone μCT images from P14 mice in transversal and sagittal orientations. Scale bar: 5 mm. (**B**) Graphical representation of the skull length, skull width, and centroid size of *Fgfr3^Asn534Lys/+^* (*n* = 9) and *Fgfr3^+/+^* (*n* = 9) male and female mice on P14. (**C**) 3D representations showing variations of skull shape relationships between *Fgfr3^Asn534Lys/+^* and *Fgfr3^+/+^* mice on P14 using Canonical Variate. (**D**) 3D representations showing variations of mandibular shape and maxillomandibular relationships between mutants and controls using PCA (*Fgfr3^+/+^* *n* = 9 *Fgfr3^Asn534Lys/+^* *n* = 9 male and female mice on P14). (**E**) Representative *Fgfr3^+/+^* and *Fgfr3^Asn534Lys/+^* skull base μCT images from P14 mice in sagittal orientation. Complete fusion of the SOS and ISS and partial fusion of IOS in *Fgfr3^Asn534Lys/+^* mice. Scale bar: 1 mm. Safranin O staining of the fused synchondrosis confirming the μCT observation. Scale bars: 200 μm. (**F**) Graphical representation of the skull base length and foramen magnum area of *Fgfr3^+/+^* (*n* = 9) and *Fgfr3^Asn534Lys/+^* (*n* = 9) P14 male and female mice. (**G**) Representative *Fgfr3^+/+^* and *Fgfr3^Asn534Lys/+^* foramen magnum μCT images from 2-week-old mice. Scale bar: 1 mm. **P < 0.01, ****P < 0.0001 by 2-tailed Student’s *t* test.

We previously demonstrated craniofacial abnormalities in an ACH mouse model (32). Consistent with this, *Fgfr3^Asn534Lys/+^* mice exhibited abnormal skull morphology on P14 (Figure 2A). Mutant mice had a dome-shaped skull compared with *Fgfr3^+/+^* mice: length (–8.85%, *P* < 0.0001), width (+3.82%, *P* = 0.0070), and centroid size (–5.34%, *P* = 0.0002) (Figure 2B and Supplemental Table 2). Geometric morphometric analyses of cranial landmark coordinates confirmed significant shape differences between *Fgfr3^Asn534Lys/+^* mice and *Fgfr3^+/+^* littermates (Procrustes distance [*d*] = 0.0926, *P* < 0.0001) (Supplemental Figure 2A and Supplemental Table 2). Overall, *Fgfr3^Asn534Lys/+^* mice displayed a more globular skull shape, with increased width and reduced length. Most of the differences were due to the size effect, as demonstrated by the decrease in the centroid size measurement in *Fgfr3^Asn534Lys/+^* mice (Figure 2, B and C, and Supplemental Figure 2A), which is a composite size measurement of the landmarks summary (33, 34). Allometry explained 73.3% of the shape variation expressed on principal component 1 (PC1) and 56.1% of the total cranial shape variation distributed along PC1 to PC17 (Figure 2C and Supplemental Figure 2A). These results suggest that *Fgfr3^Asn534Lys/+^* mice display abnormal craniofacial endochondral and intramembranous ossification. Our previous work demonstrated mandibular defects in both a mouse model (35) and patients with ACH (36). The mandible is formed by both intramembranous and endochondral ossification. Geometric morphometric analyses of maxillomandibular landmark coordinates confirmed significant shape differences between *Fgfr3^Asn534Lys/+^* mice and controls (Supplemental Figure 2B) (*d* = 0.0805, *P* < 0.001). 3D representations of the principal component analysis (PCA) result show that, in *Fgfr3^Asn534Lys/+^* mice, the mandible was prognathic, and the intercondylar and intergonial distances were relatively wide compared with controls (Figure 2D).

Our previous work showed a premature closure of the synchondroses in the *Fgfr3^Y367C/+^* mouse (37). Interestingly, skull base μCT and histological analyses revealed spheno-occipital synchondroses (SOS) and intrasphenoidal synchondrosis (ISS) fusion, and partial fusion of the intra-occipital synchondroses (IOS) in *Fgfr3^Asn534Lys/+^* mice on P14, whereas synchondroses were patent in control animals (Figure 2E). Consistent with premature synchondrosis fusion, skull base length was decreased by 21% (*P* = 0.02) and foramen magnum area by 19% (*P* < 0.0001) in *Fgfr3^Asn534Lys/+^* mice compared with controls (Figure 2F and Supplemental Table 2). Additionally, foramen magnum shape was also abnormal in *Fgfr3^Asn534Lys/+^* mice (Figure 2G). These analyses demonstrate that *Fgfr3^Asn534Lys/+^* mice have abnormal endochondral ossification that results in premature fusion of the skull synchondroses (primary cartilage joints), leading to multiple craniofacial anomalies.

### Fgfr3^Asn534Lys/+^ mice showed distinct changes in the vertebrae and IVDs.

**Figure 3:**
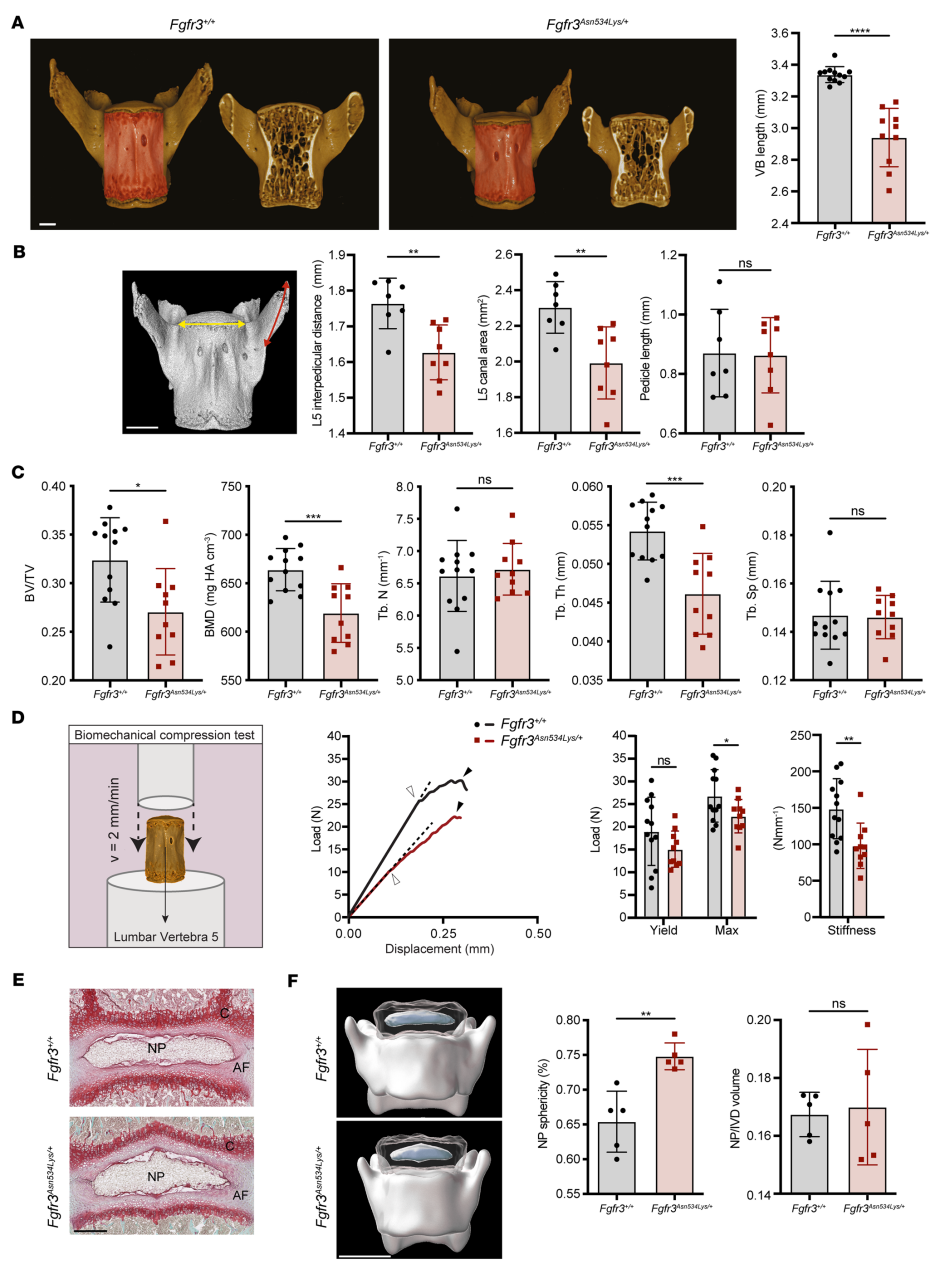
HCH mutation affects the structure of vertebral bodies and intervertebral discs. (**A**) 3D rendering of 10-week-old *Fgfr3^+/+^* and *Fgfr3^Asn534Lys/+^* male mice L5 vertebrae. In red is highlighted the ROI on the vertebral body analyzed by μCT. Scale bar: 0.5 mm. Graphical representation of the vertebral body (VB) length. (**B**) Graphical representation of L5 interpedicular distance, L5 canal area, and pedicle length of 10-week-old *Fgfr3^+/+^*(*n* = 12) and *Fgfr3^Asn534Lys/+^* (*n* = 10) male mice. On the L5 3D rendering, the interpedicular distance is highlighted in yellow, while the pedicle length is in red. Scale bar: 0.5 mm. (**C**) Dot plots for the trabecular bone parameters studied in *Fgfr3^+/+^* (*n* = 12) and *Fgfr3^Asn534Lys/+^* (*n* = 10) mice: bone mineral density (BMD), ratio of bone volume to tissue volume (BV/TV), trabecular number (Tb.N), trabecular space (Tb.Sp), and trabecular thickness (Tb.Th). NS, not significant. (**D**) Schematic representation of the setup required for lumbar vertebra compression. Representative load displacement curves (white arrow head = yield load; black arrow head = maximal load; dotted line = stiffness) and histograms for the mechanical parameters of *Fgfr3^+/+^* (*n* = 12) and *Fgfr3^Asn534Lys/+^* (*n* = 10) vertebrae: yield load, maximum load, and stiffness. NS, not significant. (**E**) Safranin O staining of lumbar intervertebral disc (IVD) (L5–L6) in P14 mice. Scale bar: 250 μm. (**F**) HREM 3D reconstruction and visualization of IVD and lumbar vertebrae. Scale bar: 1000 μm. Graphical representation of nucleus pulposus (NP) sphericity and NP/IVD volume ratio of *Fgfr3^+/+^* (*n* = 5) and *Fgfr3^Asn534Lys/+^* (*n* = 5) mice. NS, not significant; *P < 0.05, **P < 0.01, ***P < 0.001, ****P < 0.0001 by 2-tailed Student’s *t* test (**A**–**D**) or Mann-Whitney test (**F**).

We previously reported that *Fgfr3* activating mutations lead to abnormalities in both vertebrae and IVDs in an *Fgfr3^Y367C/+^* mouse model (37). For this reason, we hypothesized that the activating p.Asn534Lys mutation could affect the axial skeleton. μCT analysis of the fifth lumbar (L5) vertebrae in 10-week-old male mice demonstrated 12% reduction in vertebral body length (*P* < 0.0001) (Figure 3A), 8% reduction in interpedicular distance (*P* = 0.0037) (Figure 3B), and 14% reduction in canal area (*P* = 0.037) in *Fgfr3^Asn534Lys/+^* mice compared with *Fgfr3^+/+^* littermates. Furthermore, morphometric analysis demonstrated that L5 vertebrae from *Fgfr3^Asn534Lys/+^* and *Fgfr3^+/+^* mice differed significantly in shape on PC1 (35.98% of the total variance) and PC2 (19.86% of the total variance) (*d* = 0.0872, *P* = 0.0005) (Supplemental Figure 3A). Vertebral trabecular bone parameters showed 18% reduction in bone volume per tissue volume (BV/TV) (*P* < 0.0001), 15% decrease in trabecular thickness (Tb.Th) (*P* < 0.0001), and 7% reduction in BMD (*P* < 0.0001) in *Fgfr3^Asn534Lys/+^* compared with *Fgfr3^+/+^* mice (Figure 3C and Supplemental Table 4). Consistent with these findings, lumbar vertebrae biomechanical testing demonstrated a 22% reduction in maximal load (*P* < 0.04) and a 34% (*P* < 0.0044) decrease in stiffness in *Fgfr3^Asn534Lys/+^* compared with *Fgfr3^+/+^* mice (Figure 3D and Supplemental Table 7). These data demonstrate that *Fgfr3^Asn534Lys/+^* mice have reduced vertebral bone mass and strength, and suggest that patients with HCH may be at increased risk of vertebral fractures.

The IVD, which is a cartilaginous joint localized between vertebral bodies, is severely affected in patients with ACH (5). Thus, we examined the IVD structure on histological sections of *Fgfr3^Asn534Lys/+^* and control mice on P14. Histological analysis of the L5–L6 vertebral section, stained with Safranin O or hematoxylin and eosin (H&E), demonstrated IVD deformation with “reversed herniation” of the inner annulus fibrosus (iAF) into the nucleus pulposus (NP) region (Figure 3E). This NP “reversed herniation” was visible in histological sections from P1 to P90 (Supplemental Figure 3B). To investigate a possible direct role of FGFR3 in IVD development, we performed FGFR3 immunostaining on P14 mouse IVDs. FGFR3 is normally expressed in the NP and AF in *Fgfr3^+/+^* littermates; however, we noticed an overexpression of FGFR3 in *Fgfr3^Asn534Lys/+^*mice (Supplemental Figure 3C). To quantify the IVD deformation, we performed high-resolution episcopic microscopy (HREM). 3D rendering of the L5 vertebral body and IVD demonstrated that the NP was abnormally shaped in *Fgfr3^Asn534Lys/+^* compared with *Fgfr3^+/+^* mice (sphericity 0.75 vs. 0.65, respectively; *P* < 0.008) (Figure 3F). Altogether, these data showed that *Fgfr3^Asn534Lys/+^* mice have IVD abnormalities and suggest a prime role for FGFR3 in IVD development. Moreover, those results indicate that patients with HCH may be at increased risk of developing a degenerative disc disease.

### Impaired chondrocyte differentiation in the growth plate of Fgfr3^Asn534Lys/+^ mice.

**Figure 4:**
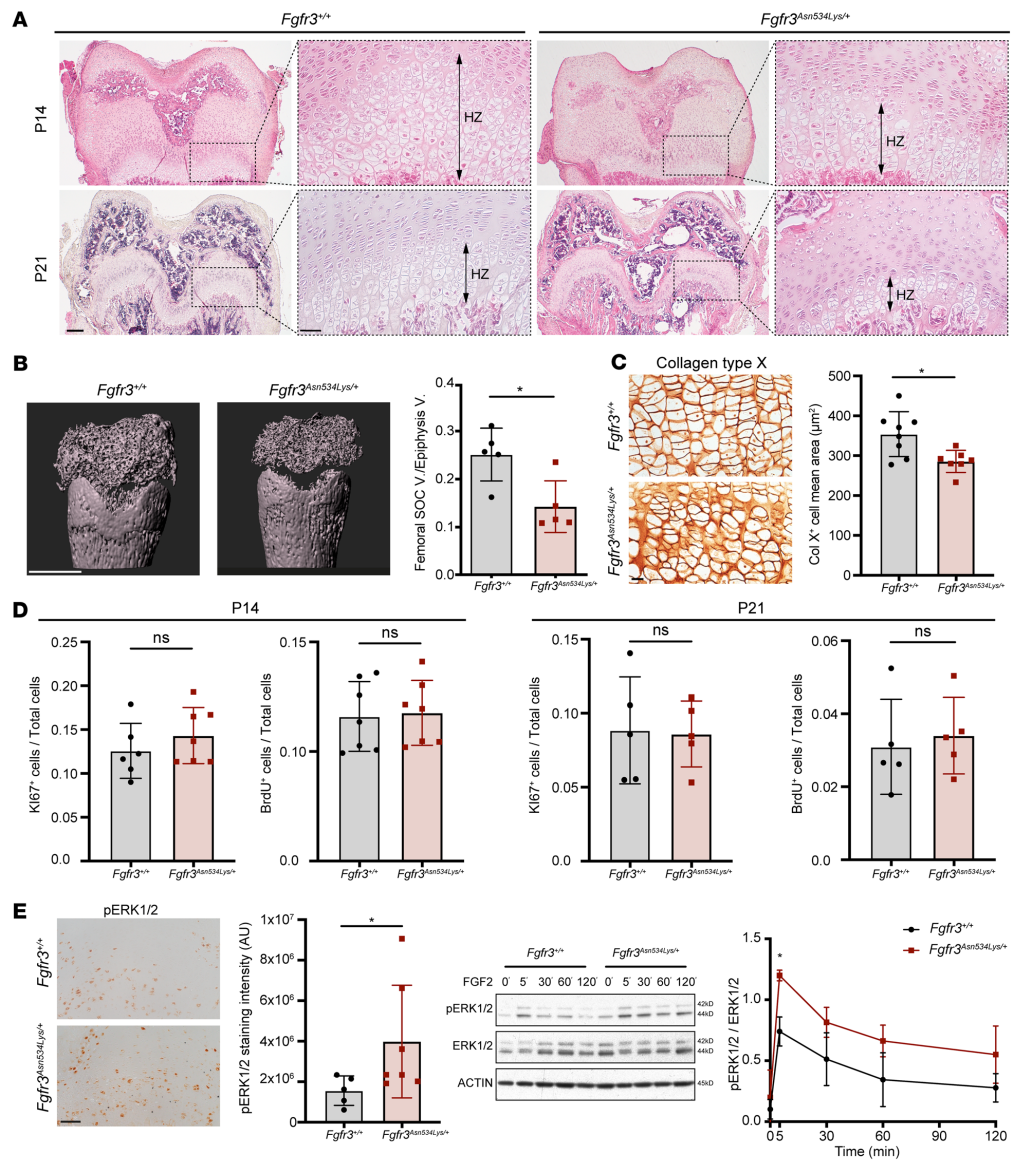
Analyses of the cartilage growth plate and the secondary ossification center (SOC). (**A**) H&E staining of femur distal growth plate on P14 and P21 showing SOC delay and reduced hypertrophic cartilage area. HZ, hypertrophic zone. Scale bars: 200 μm and 50 μm (zoomed-in images). (**B**) Left: Visualization of SOC of the distal femur. Scale bar: 1000 μm. Right: Graphical representation of the SOC/epiphysis volume ratio in *Fgfr3^+/+^* (*n* = 5) and *Fgfr3^Asn534Lys/+^* (*n* = 5) male and female mice. (**C**) Left: Collagen type X (Col X) immunostaining. Scale bar: 50 μm. Right: Graphical representation of mean cell area in Col X^+^ area in *Fgfr3^+/+^* (*n* = 7) and *Fgfr3^Asn534Lys/+^* (*n* = 6) male and female mice. (**D**) Ki67^+^ cells/total cells (DAPI^+^) on P14 in *Fgfr3^+/+^* (*n* = 6) and *Fgfr3^Asn534Lys/+^* (*n* = 7) male and female mice, BrdU^+^ cells/DAPI^+^ cells on P14 in *Fgfr3^+/+^* (*n* = 7) and *Fgfr3^Asn534Lys/+^* (*n* = 7), and BrdU^+^ cells/DAPI^+^ cells on P21 in *Fgfr3^+/+^* (*n* = 5) and *Fgfr3^Asn534Lys/+^* (*n* = 5) male and female mice. (**E**) Left: p-Erk1/2 immunostaining on P14 growth plate. Scale bar: 50 μm. Graphical representation of relative intensity of p-Erk1/2 on *Fgfr3^+/+^* (*n* = 5) and *Fgfr3^Asn534Lys/+^* (*n* = 7) male and female growth plates. Representative Western blots of p-Erk1/2 (left) and Erk1/2 in primary chondrocytes with FGF2 stimulation over time (0, 5, 30, 60, 120 minutes), from 4 independent Western blots with (*n* = 5 mice per group). Graphical representation of p-Erk1/2/Erk1/2 ratio over time. NS, not significant; *P < 0.05 by Mann-Whitney test (**B**–**D** and left graph in **E**) or 2-way ANOVA with Šidák’s multiple-comparison test (right graph in **E** [p-Erk1/2/Erk1/2 ratio over time]).

We next sought to study chondrocyte proliferation and differentiation in the cartilage growth plate. It is well known that endochondral bone formation is severely affected by *FGFR3* activating mutations and this was demonstrated in various *Fgfr3* mouse models of ACH and TD (17–19, 21). We found no obvious difference in *Fgfr3^Asn534Lys/+^* epiphyseal growth plate structures of the femur compared with controls on P7, thus confirming the absence of severe skeletal phenotype after birth (data not shown). Histological analysis of growth plate sections on P14 and P21 demonstrated progressive disorganization of the growth plate in *Fgfr3^Asn534Lys/+^* mice compared with *Fgfr3^+/+^* from P14 that was characterized by reduced hypertrophic zone width and delayed secondary ossification center (SOC) formation (Figure 4A). μCT analysis confirmed a 43% reduction in SOC volume per epiphyseal volume on P14 in *Fgfr3^Asn534Lys/+^* mice (*P* = 0.0159) (Figure 4B). To determine the underlying cellular mechanisms, we investigated chondrocyte differentiation by expression of the late-stage differentiation marker collagen type X, which demonstrated smaller hypertrophic chondrocytes due to a lack of cell swelling in *Fgfr3^Asn534Lys/+^* mice compared with *Fgfr3^+/+^* (Figure 3C) and a 20% reduction in the total area of collagen type X–positive cells (*P* = 0.0401) (Figure 4C).

By contrast, chondrocyte proliferation in *Fgfr3^Asn534Lys/+^* mice investigated by both Ki67 staining and BrdU incorporation did not differ from controls on either P14 or P21 (Figure 4D). To further examine the underlying molecular mechanisms, we analyzed Erk1/2 phosphorylation in vivo and in vitro, as MAPK activation is known to inhibit chondrocyte differentiation in ACH and TD (37–39). p-Erk1/2 immunostaining was increased by 13% in hypertrophic chondrocyte areas from *Fgfr3^Asn534Lys/+^* mice compared with *Fgfr3^+/+^* (*P* = 0.048) (Figure 4E). Consistent with this, FGF2 treatment of primary chondrocyte cultures from *Fgfr3^Asn534Lys/+^* mice resulted in increased and sustained Erk1/2 phosphorylation compared with control cells (*P* < 0.05), thus indicating that the p.Asn534Lys mutation overactivated FGFR3 and its downstream MAPK pathway (Figure 4E). These data demonstrate that anomalies of the growth plate of *Fgfr3^Asn534Lys/+^* mice are predominantly a consequence of impaired chondrocyte differentiation and hypertrophy rather than abnormal proliferation.

### Long bones from Fgfr3^Asn534Lys/+^ mice have reduced trabecular bone volume but increased cortical bone volume and mineralization.

**Figure 5:**
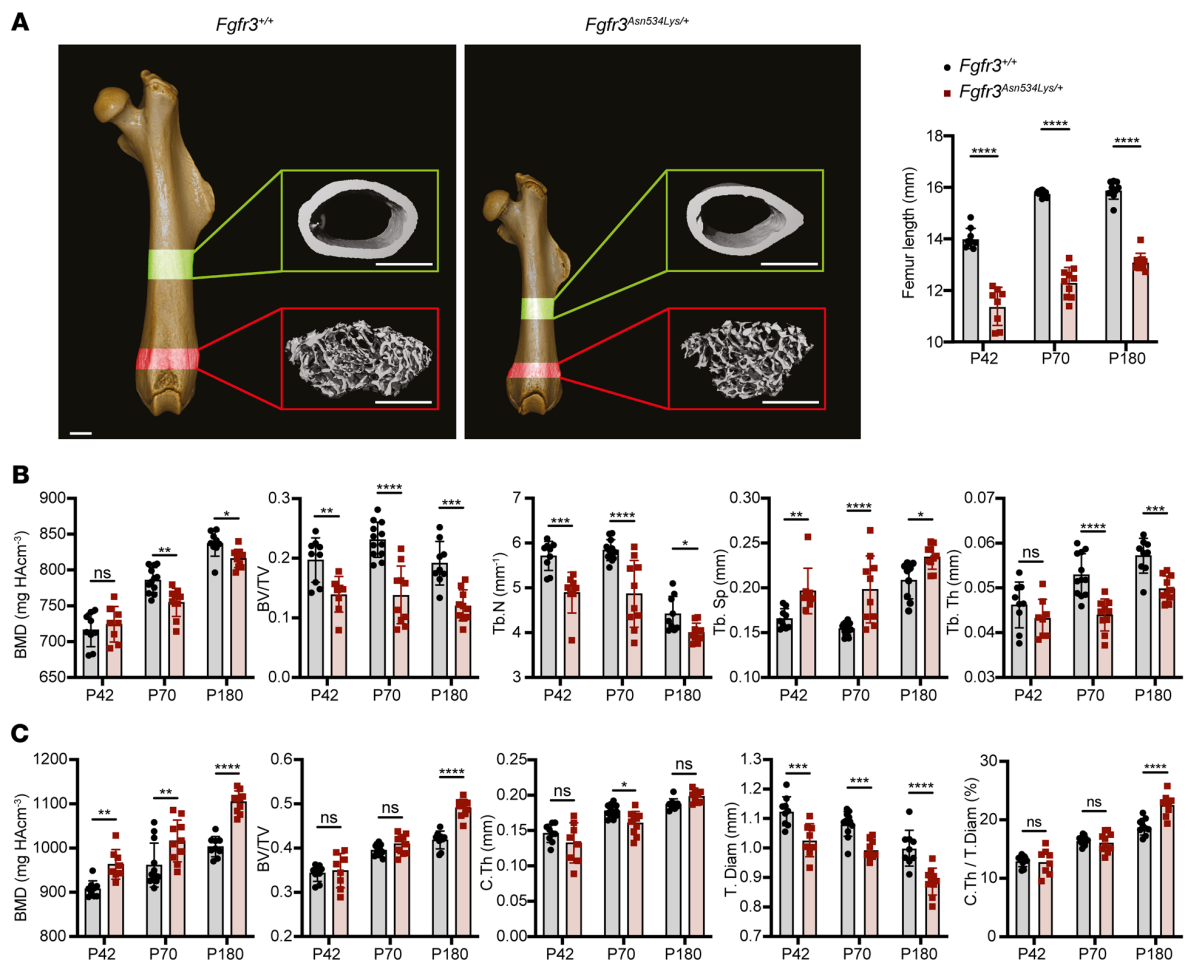
The microarchitecture of the femur is affected in *Fgfr3^Asn534Lys/+^* mice. (**A**) 3D visualization of representative P70 *Fgfr3^+/+^* and *Fgfr3^Asn534Lys/+^* male femurs. Highlighted in red is the ROI selected for the trabecular bone analyses, while the area in green indicates the ROI chosen for the cortical bone studies. Scale bars: 1 mm. Graphs show the differences in size on P42 in *Fgfr3^+/+^* (*n* = 9) and *Fgfr3^Asn534Lys/+^* (*n* = 8), P70 *Fgfr3^+/+^* (*n* = 12) and *Fgfr3^Asn534Lys/+^* (*n* = 10), and P180 *Fgfr3^+/+^* (*n* = 9) and *Fgfr3^Asn534Lys/+^* (*n* = 10) of the femurs analyzed. (**B**) Histograms for the trabecular bone parameters of P42, P70, and P180 femurs obtained by μCT: bone mineral density (BMD), ratio of bone volume to tissue volume (BV/TV), trabecular number (Tb.N), trabecular space (Tb.Sp), and trabecular thickness (Tb.Th). HA, hydroxyapatite. (**C**) Dot plots for the cortical bone parameters of P42, P70, and P180 femurs obtained by μCT: BMD, BV/TV, cortical thickness (C.Th), tissue diameter (T.Dm), and C.Th as percentage of T.Dm (C.Th/T.Dm). NS, not significant; *P < 0.05, **P < 0.01, ***P < 0.001, ****P < 0.0001 by 2-way ANOVA with Bonferroni’s post hoc test.

**Figure 6:**
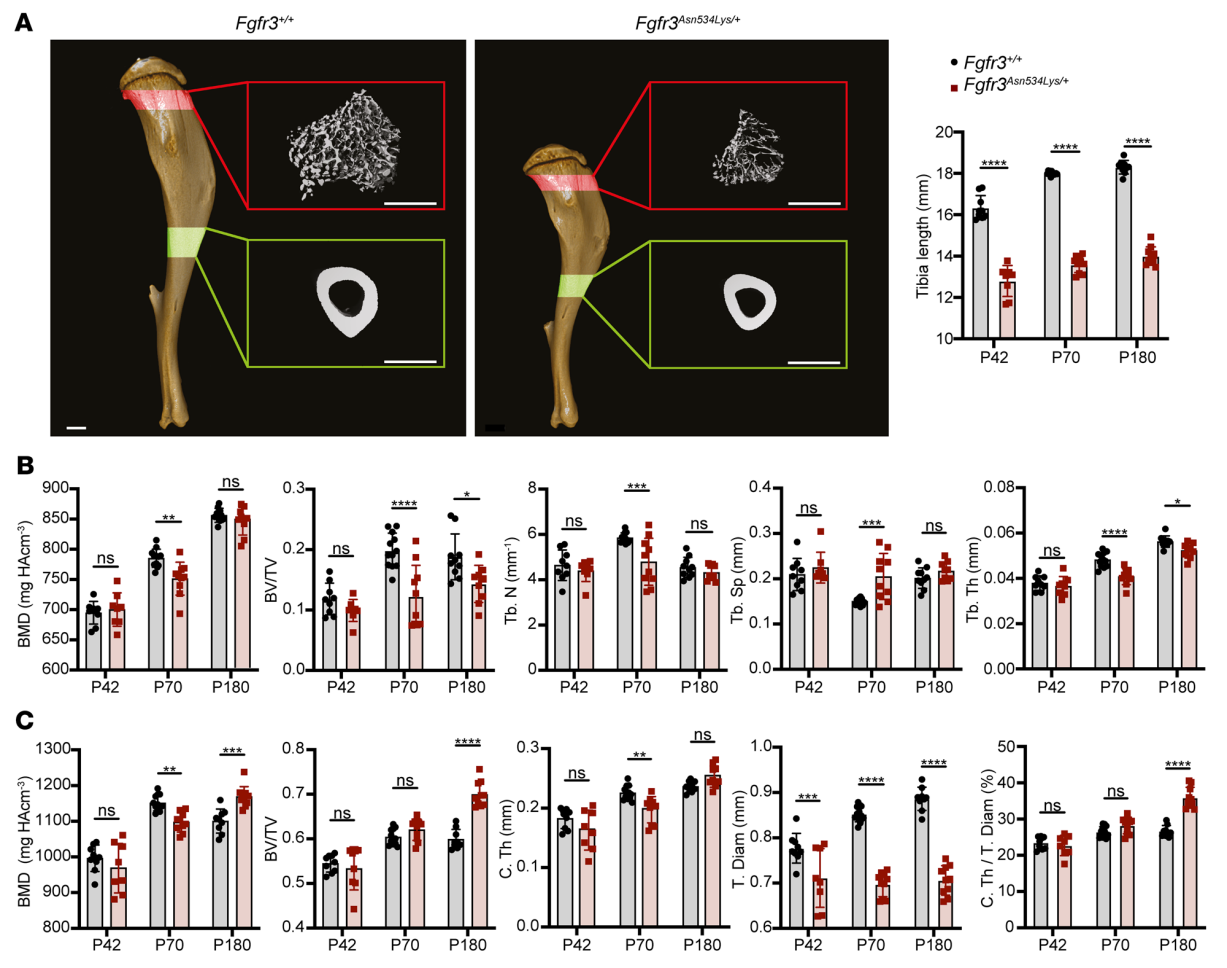
μCT analyses of the tibia in *Fgfr3^Asn534Lys/+^* mice. (**A**) 3D imaging of representative P70 *Fgfr3^+/+^* and *Fgfr3^Asn534Lys/+^* male tibiae. Highlighted in red is the ROI selected for the trabecular bone analyses, whereas the area in green indicates the ROI chosen for the cortical bone studies. Scale bars: 1 mm. The histograms show the differences in size on P42 (*Fgfr3^+/+^* *n* = 9, *Fgfr3^Asn534Lys/+^* *n* = 8), P70 (*Fgfr3^+/+^* *n* = 12, *Fgfr3^Asn534Lys/+^* *n* = 10), and P180 (*Fgfr3^+/+^* *n* = 9, *Fgfr3^Asn534Lys/+^* *n* = 10) of the tibiae analyzed. (**B**) Dot plots for the trabecular bone parameters of P42, P70, and P180 tibiae obtained by μCT: bone mineral density (BMD), ratio of bone volume to tissue volume (BV/TV), trabecular number (Tb.N), trabecular space (Tb.Sp), and trabecular thickness (Tb.Th). HA, hydroxyapatite. (**C**) Histograms for the cortical bone parameters of P42, P70, and P180 femurs obtained by μCT: BMD, BV/TV, cortical thickness (C.Th), tissue diameter (T.Dm), and C.Th as percentage of T.Dm (C.Th/T.Dm). NS, not significant; *P < 0.05, **P < 0.01, ***P < 0.001, ****P < 0.0001 by 2-way ANOVA with Bonferroni’s post hoc test.

To determine the effect of the *Fgfr3^Asn534Lys/+^* mutation in the appendicular skeleton, μCT analyses of femurs (Figure 5, Supplemental Figure 4, and Supplemental Tables 3 and 5) and tibiae (Figure 6, Supplemental Figure 5, and Supplemental Tables 3 and 6) from *Fgfr3^+/+^* and *Fgfr3^Asn534Lys/+^* mice were performed on P42, P70, and P180. Trabecular analysis of femurs showed reduced BV/TV (30%–42%, *P* = 0.0038 to *P* < 0.0002), decreased trabecular number (Tb.N) (10%–17%, *P* = 0.0007 to *P* < 0.0001), and increased trabecular space (Tb.Sp) (12%–28%, *P* = 0.0342 to *P* < 0.0001) at all ages, decreased Tb.Th (13%–17%, *P* = 0.0009 to *P* < 0.0001) on P70 and P180, and decreased trabecular BMD (4%, *P* = 0.0013) on P70 (Figure 5B and Supplemental Figure 4). BMD (*P* = 0.787) and Tb.Th (*P* = 0.4208) were not significantly different in P42 mice, indicating that the phenotype was more severe in older animals. Interestingly, none of the tibia trabecular bone measurements were different on P42, whereas results from tibia trabecular analysis were similar to those observed in femurs (Figure 6B and Supplemental Figure 4) on P70 and P180. Specifically, we observed reduced BV/TV (24%–38%, *P* < 0.0001) and Tb.Th (7%–15%, *P* < 0.001).

We also evaluated the cortical bone parameters of femoral and tibial mid-shafts (Figure 5C, Figure 6C, and Supplemental Figure 4 data from P42 to P180). Femur and tibia cortical analysis showed a decreased total diameter (8%–11%, *P* = 0.0003 to *P* < 0.0001) at all ages. Surprisingly, in the femoral bone, we observed an increase in cortical BMD (6%–10%, *P* = 0.002 to *P* < 0.0001) at all ages in mutant compared with *Fgfr3^+/+^* mice (Figure 5C and Supplemental Table 5), whereas a higher BMD was observed in tibiae exclusively in P180 (6%, *P* = 0.0077) mutants (Figure 6C). We also observed an increase in cortical BV/TV (17%, *P* < 0.0001) and in the ratio of the cortical thickness to the total diameter (C.Th/T.Dm) (4%–9%, *P* < 0.0001) on P180, for both femurs (Figure 5C and Supplemental Figure 5) and tibiae (Figure 6C and Supplemental Figure 5). Altogether, these results suggest that a high cortical BMD is a pathological feature of HCH, concordant with the lower cortical BMD observed in the *Fgfr3*-knockout model (25).

### Fgfr3^+/Asn534Lys^ mice display osteoblast and osteocyte abnormalities.

**Figure 7:**
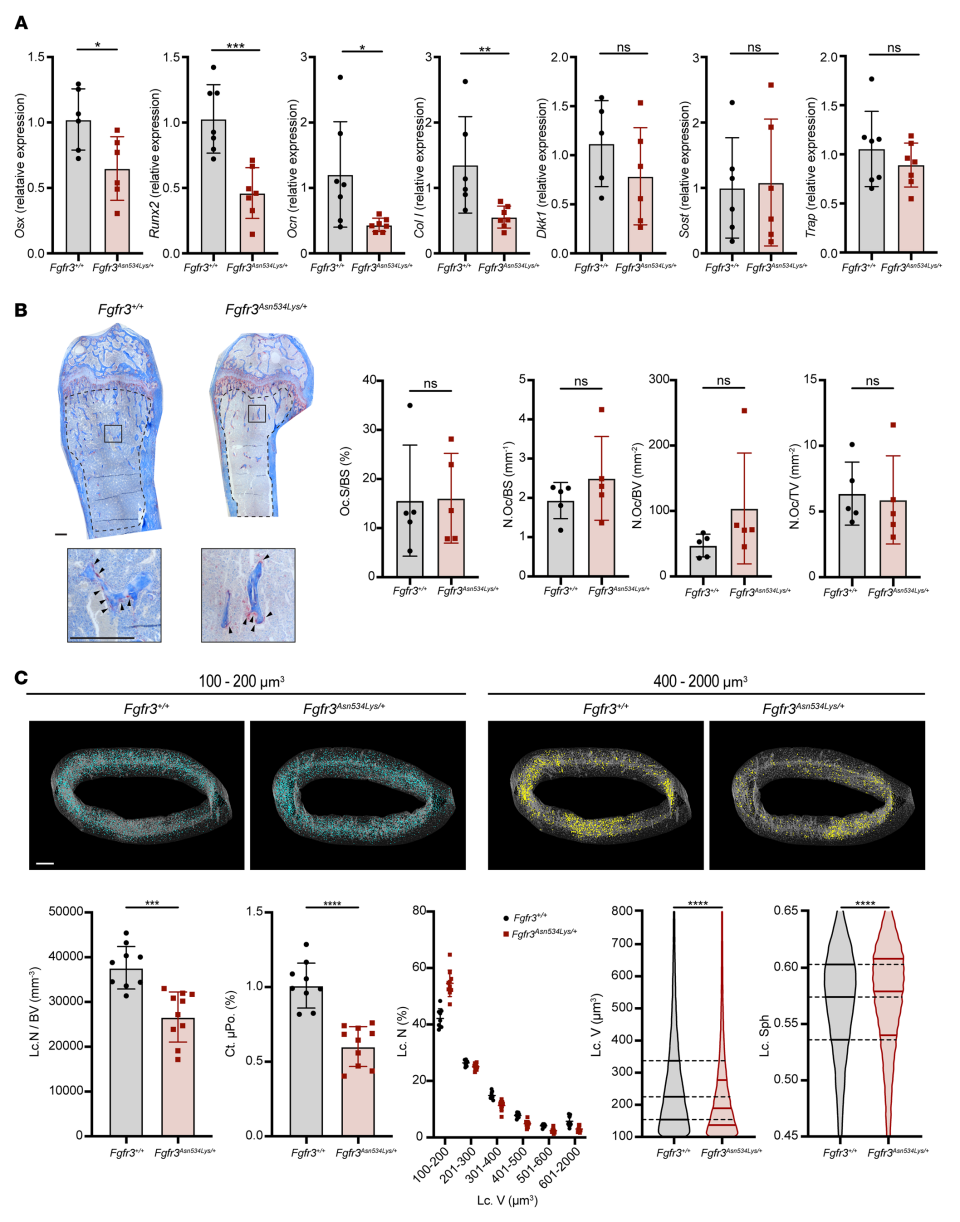
Defective bone homeostasis. (**A**) RT-qPCR analysis of femoral cortical bone. Relative expression in *Fgfr3^+/+^* (*n* = 7) and *Fgfr3^Asn534Lys/+^* (*n* = 6) male mice is shown. (**B**) Histological sections of *Fgfr3^+/+^* and *Fgfr3^Asn564Lys/+^* decalcified 2-month-old male femurs stained for tartrate-resistant acid phosphatase (TRAP). Black arrow heads indicate red TRAP-stained osteoclasts. Scale bars: 200 μm. Graphs show osteoclast surface per mm of bone surface (Oc.S/BS), number of osteoclasts per mm of bone surface (N.Oc/BS), number of osteoclasts per mm^2^ of bone (N.Oc/BV), and number of osteoclasts per mm^2^ of tissue area analyzed (N.OC/TV) (mean ± SD, *n* = 5 per genotype). Dashed lines outline the ROI of the trabecular area that was quantified. (**C**) μCT images representative of osteocyte lacunae in femoral cortical bone of 6-month-old male *Fgfr3^+/+^* (*n* = 9) and *Fgfr3^Asn564Lys/+^* (*n* = 10) mice highlighting small (100–200 μm^3^) and large lacunae (400–2000 μm^3^). Scale bar: 250 μm. The bar graphs show osteocyte lacunae number per bone volume (Lc.N/BV), percentage cortical microporosity (Ct.μPo.), and relative frequency distribution of lacunae volumes (Lc.V) in *Fgfr3^+/+^* and mutant mice. Violin plots show distribution of lacunae volumes (Lc.V) and lacunae sphericity (Lc.Sph) in *Fgfr3^+/+^* and *Fgfr3^Asn564Lys/+^* animals. The percentage of number of lacunae (Lc.N) was obtained by binning the osteocyte lacunae based on the illustrated range of volumes. NS, not significant; *P < 0.05, **P < 0.01, ***P < 0.001, ****P < 0.0001 by Mann-Whitney test (**A**, **B**, and bar graphs in **C**) or 2-tailed Kolmogorov-Smirnov test (violin plots in **C**).

To further investigate the cellular and molecular mechanisms underlying these skeletal abnormalities in *Fgfr3^+/Asn534Lys^* mice, we investigated osteoblastic and osteoclastic marker expression by performing RT-qPCR using RNA isolated from femur cortical bone. Osteoblast lineage characteristic gene expression was decreased in *Fgfr3^+/Asn534Lys^* mice compared with *Fgfr3^+/+^* mice. These genes included osterix (*Osx*) (*P* = 0.0411), runt-related transcription factor 2 (*Runx2*) (*P* = 0.0006), osteocalcin (*Ocn*) (*P* = 0.0262), and collagen type I (*Col I*) (*P* = 0.0047) (Figure 7A). By contrast, negative regulators of WNT signaling, sclerostin (*SOST*) (*P* = 0.9372) and Dickkopf Wnt signaling pathway inhibitor 1 (*DKK1*) (*P* = 0.2468), did not differ (Figure 7A).

The expression of the osteoclast marker tartrate-resistant acid phosphatase (*TRAP*) did not vary between *Fgfr3^+/Asn534Lys^* and *Fgfr3^+/+^* mice (*P* = 0.5350) (Figure 7A). Furthermore, analysis of TRAP-stained distal femur sections from *Fgfr3^+/Asn534Lys^* and *Fgfr3^+/+^* mice on P60 showed no difference in osteoclast parameters (Figure 7B). To investigate the osteocyte cortical lacunar network, we performed high-resolution μCT analyses of femur mid-shaft cortical bone from *Fgfr3^+/Asn534Lys^* and *Fgfr3^+/+^* male mice on P180. *Fgfr3^+/Asn534Lys^* mice showed a 40% decrease in cortical microporosity (Ct.μPo.) (*P* < 0.0001), 29% decrease in lacunae number per bone volume (Lc.N/BV) (*P* = 0.0002), and 16% decrease in median lacunae volume (Lc.V) (*P* < 0.0001) compared with *Fgfr3^+/+^* (Figure 7C).

The distribution of lacuna sizes also differed in mutant mice displaying a higher percentage of small lacunae (100–200 μm^3^: 55% in *Fgfr3^+/Asn534Lys^* vs. 41% in *Fgfr3^+/+^*) and a lower percentage of large lacunae (400–2000 μm^3^: 10% in *Fgfr3^+/Asn534Lys^* vs. 15% in *Fgfr3^+/+^*) (Figure 7C). Furthermore, the lacuna shape sphericity (Lc.Sph) was increased in mutant mice, with a median of 0.579 in *Fgfr3^+/Asn534Lys^* versus 0.574 in *Fgfr3^+/+^* mice (*P* < 0.0001) (Figure 7C). Together, these data demonstrate that the *Fgfr3* p.Asn534Lys mutation results in abnormal osteoblast and osteocyte functions that lead to contrasting effects in trabecular and cortical bone.

### Long bones from Fgfr3^Asn534Lys/+^ mice have reduced strength and stiffness.

**Figure 8:**
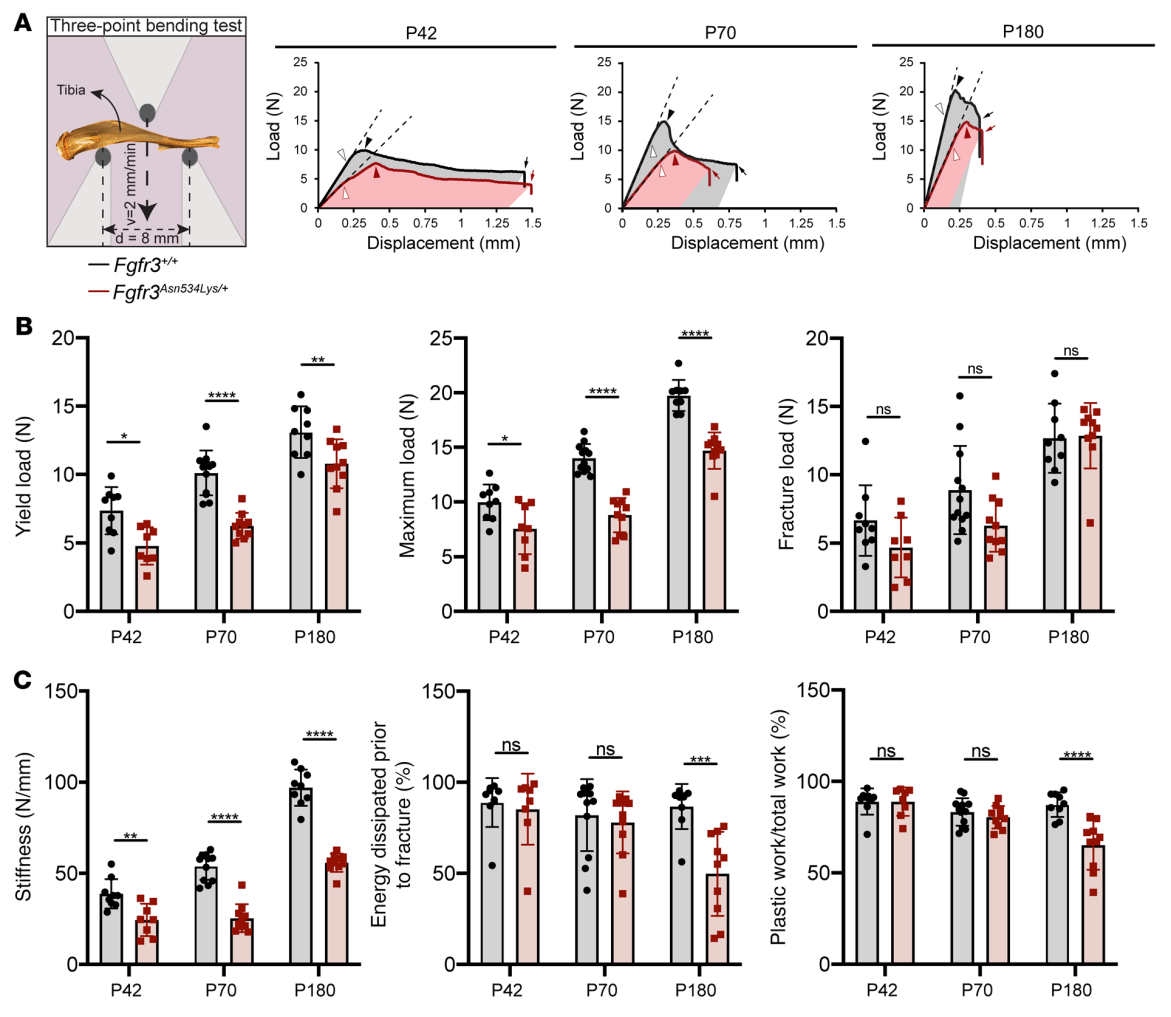
Evaluation of bone strength in tibiae. (**A**) Schematic representation of a 3-point bend test of tibiae and representative load displacement curves of tibiae from male P42, P70, and P180 *Fgfr3^+/+^* and *Fgfr3^Asn534Lys/+^* mice (white arrowhead = yield load; black arrowhead = maximal load; black arrow = fracture load; dotted line = stiffness; colored area under curve = energy prior to fracture). (**B**) Histograms for the biomechanical parameters of *Fgfr3^+/+^* (P42 *n* = 9, P70 *n* = 12, P180 *n* = 9) and *Fgfr3^Asn534Lys/+^* (P42 *n* = 8, P70 *n* = 10, P180 *n* = 10) tibiae of male mice: yield load, maximal load, and fracture load. (**C**) Stiffness, percentage of energy prior to fracture (toughness), and plastic work as percentage of total work. NS, not significant. *P < 0.05, **P < 0.01, ***P < 0.001, ****P < 0.0001 by 2-way ANOVA with Bonferroni’s post hoc test.

To determine the effect of the FGFR3 p.Asn534Lys mutation on long bone strength, 3-point bend testing of tibiae (Figure 8 and Supplemental Table 7) from *Fgfr3^+/+^*and *Fgfr3^Asn534Lys/+^* mice was performed on P42, P70, and P180. Biomechanical analysis showed reduced yield load (18%–38%, *P* = 0.0077 to *P* < 0.0001), maximal load (24%–37%, *P* = 0.0126 to *P* < 0.0001), and stiffness (37%–53%, *P* = 0.0014 to *P* < 0.0001) at all ages, and reduced toughness (energy dissipated at fracture) (37%, *P* < 0.0001) and plastic work to total work (21%, *P* < 0.0001) on P180. These data demonstrate that long bones in young *Fgfr3^Asn534Lys/+^* mice are weak and flexible. In older animals, the increased cortical thickness and mineralization results in reduced toughness and a brittle phenotype. Of particular interest, the latter finding demonstrated that a higher cortical BMD in 6-month-old tibiae was detrimental to their biomechanical performance.

## Discussion

HCH is the mildest form of FGFR3-related chondrodysplasia. It has been reported that patients who have the most frequent mutation (Asn540Lys) have a more severe form of dwarfism (8, 40–42). Currently, little is known about bone phenotype and growth in children with HCH. Various studies have described HCH patients with short stature, body disproportion, and relative macrocephaly (43). Our study was designed to elucidate the impact of the most common HCH mutation (Asn540Lys) on skeletal development and adult bone maintenance and strength, as this has not been systematically characterized to the best of our knowledge. This was achieved through the generation of the *Fgfr3^Asn534Lys/+^* mouse model expressing the Asn534Lys gain-of-function mutation.

FGFR3 regulates a wide range of physiological processes and its aberrant signaling has been linked to dwarfism (14), overgrowth (44), and several types of malignancies (45–47). Analysis of the kinase activity of selected FGFR3 variants harboring missense mutations showed that the Asn540Lys mutation resulted in a large increase in autophosphorylation (48). We confirmed enhanced activation of this variant and the MAPK pathway in vitro using primary chondrocytes isolated from the *Fgfr3^Asn534Lys/+^* rib cages and in vivo with p-Erk1/2 immunostaining.

We attempted to elucidate some HCH phenotypic traits. First, the dwarf phenotype observed in the *Fgfr3^Asn534Lys/+^* mutant is more pronounced in older animals, which is similar to the human pathology (8, 49). We confirmed that the activating HCH mutation modified *Fgfr3^Asn534Lys/+^* mouse skull shape by inducing macrocephaly and prognathism. Interestingly, we observed a significant decrease in the foramen magnum area due to a premature fusion of the synchondroses, which has also been observed in another FGFR3 gain-of-function mouse model (37). These findings are consistent with previous data demonstrating that FGFR3 activation and MAPK downstream signaling in chondrocytes promotes foramen magnum stenosis (50). Overall, these data identify important craniofacial abnormalities in *Fgfr3^Asn534Lys/+^* mice and suggest that similar anomalies may be present in patients with HCH.

During growth, we observed defects of the axial skeleton, with structural anomalies of the IVDs. Concomitant low BMD of the lumbar vertebrae is also observed at adult stages. Interestingly, the loss of normal IVD architecture suggests that these anomalies are due to the key role of FGFR3 in controlling IVD development, with an impact on NP and AF formation during embryonic development. Recently, FGFR3 has been described to be indispensable to NP development and proliferation (51). This phenotypic trait has not been described in HCH and could be further clinically explored.

As expected, robust FGFR3 activity impaired the endochondral ossification, thus inducing a dysregulation of chondrocyte differentiation. The absence of visible defective proliferation during the postnatal period confirms that the reduced bone growth in the *Fgfr3^Asn534Lys/+^* mouse model is mostly due to hypertrophic chondrocyte enlargement impairment modulated by increased Erk1/2 activation (52, 53). In the future, it will be relevant to investigate whether *Fgfr3^Asn534Lys/+^* hypertrophic chondrocytes undergo apoptosis and autophagic processes (54) and/or transdifferentiate into osteoblasts (55).

Studies concerning FGFR3-related chondrodysplasias have mostly focused on growth plate cartilage from birth to puberty in young animals. Growth plate cartilage plays a central role in the proper development and growth of endochondral bone. During the endochondral ossification process an initial cartilage template is systematically replaced by bone. Here, one of our concerns was to decipher the bone formation during the adult period in the *Fgfr3^Asn534Lys/+^* mouse model. It is well known that bone shape and composition change throughout growth development, but no analysis to our knowledge has been done in adult mouse models. Previously, long bone low BMD and increased number of osteoclasts (27) in young animals expressing an *Fgfr3* gain-of-function mutation (G369C/+) was reported. However, inactivation of *Fgfr3* in mice showed osteopenia, reduced cortical thickness, and an increased number of osteoclasts (25).

In contrast, *Fgfr3* deletion restricted to chondrocytes leads to increased bone mass by upregulating osteogenesis and inhibiting osteoclastogenesis (56). Finally, *Fgfr3* deficiency in osteoclast lineage cells leads to increased bone mass and impaired bone regeneration by inhibiting osteoclast bone resorption activity (57). Taking advantage of the ubiquitous expression of the *Fgfr3* pAsn534Lys gain-of-function mutation in *Fgfr3^Asn534Lys/+^* mice, we deeply analyzed the bone phenotype and demonstrated that *Fgfr3* activation did not modify osteoclast differentiation; however, it decreased BMD and reduced BV/TV in the femur trabecular bone. Moreover, bone-formation modifications were less obvious for the tibia. Femur mineralization is highly affected from 6 weeks (P42) to 6 months (P180) of age in mutant compared with control. However, we observed a less established bone phenotype in tibiae between 2 months and 6 months of age, demonstrating that the proximo-distal differentiation of limb segments influenced distal trabecular bone formation. These data could be explained by the human pathology describing HCH as rhizomelic dwarfism. As observed in long bones, we noted a BMD decrease in vertebrae. While performing compression tests on the lumbar vertebral bodies, we revealed a trabecular bone fragility in *Fgfr3^Asn534Lys/+^* mice. These findings with the presence of IVD anomalies should be compared to the human pathology. The low BMD we observed in *Fgfr3^Asn534Lys/+^* mice is concordant with low lumbar BMD reported in patients with HCH (58).

In long bones, it is well known that cortical and trabecular bone should be considered as 2 separate bone compartments. Trabecular bone derives directly from the growth plate, whereas cortical bone derives from periosteum mesenchymal cells that differentiate into osteoblasts. Here, we studied femur and tibia cortical bone. Surprisingly, we observed in *Fgfr3^Asn534Lys/+^* mice that cortical bone BMD and BV/TV were significantly increased for both femurs and tibiae. This increased cortical mineralization of tibiae and femurs was more pronounced at 6 months of age, but as described for the trabecular bone, the phenotype was more severe in femurs than in tibiae. To provide unique insights and valuable information regarding cortical bone in *Fgfr3^Asn534Lys/+^*, we performed a biomechanical 3-point bending test on tibiae and we observed a higher fragility in *Fgfr3^Asn534Lys/+^* tibiae than in *Fgfr3^+/+^*. Considering these findings, we confirmed that trabecular bone formation is partly regulated by growth plate and paracrine actions on osteogenesis, whereas the cortical bone is under the control of cells coming from the periosteum. Based on our findings, we can speculate that trabecular bone formation is mostly regulated by growth plate and paracrine mechanisms, while cortical bone development is primarily controlled by periosteal cells. The exact role of FGFRs in the regulation of osteocytes survival or apoptosis is poorly understood. Recently, the role of *Fgfr1* was reported in mouse models lacking *Fgfr1* alone or *Fgfr1/Fgfr2* in mature osteoblasts, showing a dramatic bone mass increase. The authors suggested that the viability/death of osteocytes was controlled by *Fgfr1* (24). Interestingly, a volumetric BMD (vBMD) study in patients with ACH showed higher cortical vBMD and lower trabecular vBMD in the tibia, confirming our data obtained from the *Fgfr3^Asn534Lys/+^* mouse model (59).

Here, our data suggest that the *Fgfr3* gain-of-function mutation deregulates osteoblastic function by promoting defective cortical bone mineralization associated with impairment of bone markers, and conversely by decreasing trabecular bone mineralization. High-resolution analyses of lacunae demonstrated the impact of the *Fgfr3^Asn534Lys/+^* mutation on osteocytes. Therefore, we can reasonably hypothesize that deregulated osteocytes may play a key role in cortical bone abnormal mineralization. As a result of altered osteoblastic lineage regulation, the osteogenesis is defective and leads to a weak and brittle bone. These various dysfunctions of osteoblast lineage in long bones seem to be more pronounced with aging. We can hypothesize that this increase in cortical bone mass observed in our *Fgfr3* gain-of-function mouse model could be associated with a decreased expression of *Fgfr1* in mature osteoblasts. It is likely that the dysregulation of *Fgfr1* could be more important in cortical bone than in trabecular bone. Future studies will be required to determine the relative contribution and the distinct roles of *Fgfr1*, *Fgfr2*, and *Fgfr3* in bone growth regulation. In our study, the *Fgfr3^Asn534Lys/+^* mutation downregulates osteoblastogenesis and not osteoclastogenesis, and results in defective osteogenesis characterized by increasing bone apposition for cortical bone and decreasing bone formation for trabecular bone. On the one hand, *fgfr3* loss-of-function downregulates suture formation in a zebrafish model, with extracellular matrix dysregulation (26). FGFR3 can moderate osteoblastogenesis through activation of downstream signaling pathways that are known to be involved in osteoblastogenesis. *Stat1*-null mice present a higher cortical BMD and dysregulated *Fgfr3* expression in osteoblasts (60). Recent research has revealed that *Fgfr3* is among the genes that distinguish the osteocyte transcriptome from that of other cells (61) and confirm its gene expression in both femoral and tibial osteocytes. In addition, it was shown that depletion of genes in the osteocyte transcriptome (*Cttnbp2*, *Ldlrad4*, and *Auts2*) can result in anomalies of the lacuno-canalicular network in cortical bone. Those defects can be detected by changes in the size and shape of the lacunae. Similarly, in our *Fgfr3^Asn534Lys/+^* model, we observed modifications in both size and sphericity of the lacunae, which may suggest a direct role of FGFR3 in cortical osteocytes.

Finally, other studies indicating that defective cortical bone formation may be under the control of MAPK and the suppressor of cytokine signaling (SOCS) revealed that FGFR3 and SOCS3 are tightly linked in bone maturation (62). These data may be consistent with our present findings suggesting that FGFR3 overactivation by gain-of-function mutation can lead to SOCS3 overexpression, thus inducing higher corticalization in long bone. Additional studies are needed to fully elucidate and distinguish the role of FGFR3 in trabecular and cortical bone. Lastly, it is difficult to dissociate cartilage from bone formation, since some hypertrophic chondrocytes have the ability to transdifferentiate into osteoblasts and further into more mature osteocytes over time (63, 64). In the context of FGFR3-related disorders, we can hypothesize that the osteoblasts derived from the abnormal hypertrophic chondrocytes are less active, thus explaining the low BMD in trabecular bone. Indeed, our findings show that *Fgfr3^Asn534Lys/+^* bone has some characteristics of osteoporotic bones, suggesting that patients with HCH may present osteoporotic traits with a higher risk of fractures in old age. Interestingly, recent studies described osteoporosis in patients with ACH and HCH (58, 65). However, other studies need to be conducted in a larger series of adult HCH patients to confirm those clinical observations. Due to the differing mineralization densities detected in trabecular and cortical bone, special attention will be demanded in designing a pharmacological treatment of osteoporosis for elderly patients with HCH.

These data highlight the deleterious effect of FGFR3 gain-of-function mutation on long bone modeling during development and aging, which may have a major impact on the management of a health condition, such as osteoporosis that weakens bones, in adult patients with HCH.

## Methods

### Generation of Fgfr3^Asn534Lys/+^ mice.

A targeting vector was constructed containing the Asn534Lys (corresponding to Asn540Lys in humans) mutation in exon 12 of *Fgfr3* and the *loxP*-flanked *neo*/STOP cassette (neomycin intron 10). The excision of the *loxP* cassette of the mouse line *Fgfr3^+/^^loxp^ ^Asn534Lys^* was achieved using the CMV-Cre mouse line (31). *Fgfr3^loxAsn534Lys^* ES cells (SY05) were injected into blastocysts from gray C57BL/N mice by Polygene. The resulting chimeras were bred with C57BL/6N mice to obtain *Fgfr3^loxAsn534Lys/+^* mice.

Genomic DNA was isolated from tails using a NucleoSpin Tissue kit (Macherey-Nagel) according the manufacturer’s instructions. The mice were genotyped using 3 sets of primers: 5′-GTGGGGGTTCTGCGGTTGG-3′ and 5′-TGACAGGCTTGGCAGTACGG-3′ targeting the *Fgfr3* gene to distinguish between WT (303 bp) and mutant mice (421 bp); 5′-AACCTCTACAAATGTGGTATGGCTG-3′ and 5′-CCGCTTCCTCGTGCTTTACGGTA-3′ to isolate the construct by targeting the neomycin box in the *Fgfr3* gene construct; and 5′-AGCGTTTTCGTTCTGCCAAT-3′ and 5′-ACCCTGTTACGTATAGCCGAAATT-3′ for *cre*. For all analyses, the second generation of mice was used to obtain WT littermates as controls and to remove Cre from the first generation of mice. Both male and female mice were used for longitudinal analyses. On P7, P14, and P21, *Fgfr3^Asn534Lys/+^* and *Fgfr3^N534K/+^* mice received intraperitoneal injection of BrdU labeling reagent (00-0103, Invitrogen) at 10 μL/g body weight, 2 hours before sacrifice. Long bones were measured by caliper (VWRi819-0013, VWR International).

### X-ray microradiography.

The patients and their families were evaluated by a medical geneticist. The research was conducted ethically in accordance with the World Medical Association Declaration of Helsinki. Written informed consents were obtained from the patient’s legal guardian/parent for publication of x-rays.

X-ray radiography imaging of the fetus was performed immediately postmortem using a Faxitron UltraFocus digital x-ray radiography system (Faxitron Bioptics). Ventral images were taken at 1×.

### Sequence analysis.

The exons were amplified with primers 5′-TAAGCCTCTTGGAGAAGGCTGCTTTG-3′ and 5′-CATTCCCTAGCTCAGGCAAAC-3′ targeting exon 12 of *Fgfr3* containing the C1602A mutation. Amplification products were purified by ExoProStar 1-Step (Amersham) and directly sequenced with the Big Dye Terminator v3.1 Cycle Sequencing Ready Reaction kit (Applied Biosystems) on an automatic sequencer (3500xL, Applied Biosystems). Sequence analyses were performed with Sequencing 6 software (Applied Biosystems).

### Whole-body alizarin red and Alcian blue staining.

P1 mice were skinned, fixed in 95% ethanol, and stained with Alcian Blue 8GX and Alizarin Red (Sigma-Aldrich). Whole skeletons were cleared by potassium hydroxide treatment and stored in 100% glycerol according to standard protocols (66). Measurements were performed using ImageJ software (67, 68).

### Morphometric analyses.

The samples consist of 9 P14 *Fgfr3^N534K/+^* mice and 9 *Fgfr3^+/+^*. μCT imaging of the skull and mandibles was performed using a Skyscan 1172 (Bruker). Samples were fixed in 70% ethanol and scanned with settings of 80 kV, 100 μA, 17.99 μm pixel resolution, and 0.5° rotation steps with a 0.5 mm aluminum filter. 3D reconstructions were generated using NRecon software (Skyscan). Visual adjustments of the cortical bone density threshold were done for each CT scan, without smoothing factors to maintain the correct contours and avoid any loss of information concerning anatomical variations. The 3D surface mesh was obtained using Avizo software (Thermo Fisher Scientific) for each specimen and then used for landmark positioning.

Landmark 3D coordinates were analyzed by geometric morphometric methods, including standardization for position, scale, and orientation through Procrustes superimposition (69). Positions of the landmarks are provided in Supplemental Figure 2A. Error measurement was assessed with Procrustes ANOVA and showed no significant differences in landmark positioning, allowing valid comparative analyses of shape variation among specimens. After Procrustes superimposition, the resulting 3D Procrustes shape coordinates were evaluated by PCA. Wire frames were used to visualize the shape differences corresponding to the skull associated with specific scores on PC1. Centroid size, the square root of the sum of squared distances of the landmarks from their centroid, was used as a proxy for size (70). The influence of size on shape (i.e., allometry) was tested with multiple multivariate regression. Procrustes distances (*d*) separating groups in the morphospace were computed and significance was assessed using permutation tests (10,000 permutation rounds). A significant value of *d* was interpreted as significant shape differences between the 2 compared groups. The geometric morphometric analyses were performed with MorphoJ v. 1.06 (71).

Foramen magnum area was evaluated using the Radinsky formula (1/4π × mediosagittal foramen magnum distance × transversal foramen magnum distance) (72). Linear skull measurements were performed using ImageJ software (67, 68). For mandibular morphometry analyses, 30 anatomical landmarks (15 per mandible, including the incisors) were defined in order to model the shape of the mandible and were placed using Avizo by the same author. In addition, 10 anatomical landmarks were defined on the maxilla (including incisors and nasal bones (5 per side) to better analyze the relationship between the maxilla and the mandible. In total, 40 landmarks were placed for each specimen. Positions of the landmarks are provided in Supplemental Figure 2B. To confirm statistically significant shape differences, ANOVA was performed considering the genotype as classifier. Vertebral morphometric analyses were performed on P70 mice (*Fgfr3^+/+^*, P70 *n* = 12; *Fgfr3^Asn534Lys/+^*, P70 *n* = 10). Images were acquired in 70% ethanol using a Scanco μCT 50 scanner (Scanco Medical; scans were performed at 70 kV, 200 μA, 0.5 mm aluminium filter). 3D surface mesh generation and 32-landmark positioning for each specimen were performed with Avizo software. Positions of the landmarks are provided in Supplemental Figure 3A. The influence of size on shape (i.e., allometry) was tested with multiple multivariate regression. Procrustes distances separating groups in the morphospace were computed and significance was assessed using permutation tests (10,000 permutation rounds).

### HREM.

After dissection, samples were fixed in 4% paraformaldehyde for 24 hours. Fixed tissues were rinsed twice with PBS and dehydrated through a graded series of methanol baths (20%, 30%, 40%, 50%, 60%, 70%, 80%, 90%, and 95%) for 4 hours. After dehydration, the samples were embedded in methacrylate resin (JB-4, Polysciences) containing eosin and acridine orange as contrast agents (73). Images of the surface of the resin block were acquired repeatedly after removal of a 1.56-μm-thick section using an optical high-resolution episcopic microscope (Indigo Scientific). The 3D images acquired by HREM were analyzed using Imaris software (Bitplane). For HREM analyses, P14 mice were used (*Fgfr3^+/+^ n* = 5, *Fgfr3^Asn534Lys/+^* *n* = 5 both male and female mice).

### Western blots.

Primary chondrocytes from P1 *Fgfr3^Asn534Lys/+^* ribs and control littermates were harvested as previously described (38). Cells were serum depleted for 24 hours and then supplemented with FGF2 (100 ng/mL) (FGF-basic, Peprotech). Chondrocyte lysates were prepared using RIPA buffer supplemented with protease inhibitor (cOmplete Mini, EDTA-free, Roche). Proteins were incubated and gently mixed on a tube rotator at 11 rpm for 2 hours at 4°C. Proteins were isolated by centrifugation at 13,000*g* for 20 minutes at 4°C, and then denatured with 2.5% β-mercaptoethanol at 95°C for 10 minutes. Extracted proteins were electrophoresed in NuPAGE 4%–12% (Life Technologies) gels and transferred onto PVDF membranes (Millipore). Blots were probed with primary antibodies rabbit anti–p-Erk1/2 (Cell Signaling Technology, 4370; 1:2,000), mouse anti–total Erk1/2 (Cell Signaling Technology, 4696; 1:1,000), and mouse anti-actin (Millipore, MAB1501; 1:5,000). Proteins of interest were detected with HRP-conjugated antibodies (Cell Signaling Technology, 7074 and 7076; 1:10,000) and visualized with ECL (Thermo Fisher Scientific). The obtained immunoreactivities were determined using ImageJ software with the “Gels and Plot lanes” plug-in. See complete unedited blots in the supplemental material.

### RT-qPCR.

Cortical bones were isolated from P70 male mice femurs. Bone marrow was flushed. Cortical bones were placed in RNAlater (Invitrogen) for 24 hours at 4°C, and then immersed in liquid nitrogen and crushed. TRIzol reagent (Invitrogen) was used to separate DNA from RNA, which was then purified with a Qiagen RNeasy Mini kit following the manufacturer’s instructions. RNAs (200 ng each) were converted into cDNA using the SuperScript III First-Strand System (Invitrogen). Quantitative real-time PCR expression analysis was performed using a 7300 Real-Time PCR system (Applied Biosystem) and Absolute SYBR Green ROX mix (Abgene). Details of the sets of primers used are available in Supplemental Table 8.

### Histology.

All bones were collected in 4% paraformaldehyde, fully decalcified in 0.5 M EDTA (pH 8.0), and paraffin embedded. Serial sections (5 μm) were used for H&E, Safranin O staining, or immunochemistry. For immunohistochemical assessment, sections were labeled with the following antibodies and a Dako Envision Kit (Dako North America, Inc): anti–collagen type X (1:100; 1-CO097-05, Quartett), anti–p-p44/42 MAPK (1:100; 4370, Cell Signaling Technology), and anti-FGFR3 (1:100; F0425, Sigma-Aldrich).

For BrdU immunostaining, antigen retrieval was performed using boiling citrate buffer (pH 6.0) and DNA denaturation was performed using 2 mol/L HCl for 30 minutes at 37°C. Primary antibody incubation with rat anti-BrdU antibody (1:1,000; ab6326, Abcam) was performed overnight at 4°C. Immunostaining visualization was performed using anti-rat–Alexa Fluor 594 antibody (Invitrogen) and counterstaining with DAPI (Prolong Gold antifade reagent, Invitrogen). Images were captured with an Olympus PD70-IX2-UCB microscope. Labeled cells were counted using ImageJ software (67, 68).

Osteoclasts were quantified on paraffin sections deparaffinized in xylene (Merck) and stained for TRAP activity. Images were acquired on a Leica DM LB2 microscope. For each sample, osteoclasts were quantified in an equivalent region of interest (ROI) in trabecular bone.

### πCT analyses and imaging of long bones and vertebrae.

Mouse male femurs, tibiae (*Fgfr3^+/+^*: P42 *n* = 9, P70 *n* = 12, P180 *n* = 9; *Fgfr3^Asn534Lys/+^*: P42 *n* = 8, P70 *n* = 10, P180 *n* = 10), and vertebrae (*Fgfr3^+/+^*: P70 *n* = 12, *Fgfr3^Asn534Lys/+^*: P70 *n* = 10, P180 *n* = 10) images were acquired in 70% ethanol using a Scanco μCT 50 scanner (scans were performed at 70 kV, 200 μA, 0.5 mm aluminium filter).

In femurs, cortical bone parameters were determined at 10 μm voxel resolution of an ROI equal to 6%–7% of the femur length centered in the mid-shaft bone 56% along the proximo-distal length of the femur, and trabecular parameters were obtained at 5 μm voxel resolution in an ROI (8%–9.5% of the femur length in size) beginning 100 μm proximal to the distal growth plate.

Similarly, in tibiae, values for the compact bone were obtained at 10 μm voxel resolution of a ROI equivalent to 5.5%–6% of the tibia length centered at 50% along the length of the tibia, and trabecular parameters were determined at 5 μm voxel resolution in an ROI corresponding to 5.5%–6% of the tibia length located at 100 μm from the end of the proximal growth plate.

Trabecular bone parameters of L5 vertebrae 5 were determined at 5 μm voxel resolution by analyzing the vertebral body region delimited by the 2 cartilage endplates. More details on ROI selection are presented in Supplemental Table 3.

Analyses and 3D rendering for trabecular and cortical bone were performed using Scanco μCT v6.1 software Evaluation and 3D Display programs (Scanco Medical). Whole-bone 3D imaging was produced with Drishti v2.6.5 (National Computational Infrastructure National Facility, The Australian National University, Canberra, Australia).

### μCT analysis of osteocyte lacunae.

Osteocyte lacuna parameters and images were obtained by μCT using a Scanco μCT 50, as mentioned above. Scans of a 0.25-mm ROI centered on the cortical bone midshaft at 56% along the proximo-distal length were run at a voxel resolution of 1 μm. μCT data were reconstructed using a segmentation threshold of 350 (851.7 mg hydroxyapatite/cm^3^) for all samples (male P180 *Fgfr3^+/+^*: *n* = 9, *Fgfr3^Asn534Lys^*: *n* = 10). DICOM images of pores and cortical bones were produced with the Scanco Image Processing Language. Stack images were processed using Fiji (68) and analyses of lacunae volumes (Lc.V) were performed with BoneJ tool Particle Analyzer (74). Pores with volumes less than 100 μm^3^ and greater than 2000 μm^3^ were excluded from the analyses in accordance with previous studies (61, 75). The sphericity of the particles (Lc.Sph) was determined using the 3D Shape Measure plugin of Fiji (76). 3D rendering of the osteocyte lacunae was obtained using the Volume Viewer plugin of Fiji.

### Biomechanical testing.

Tibial 3-point bend testing (*Fgfr3^+/+^*: P42 *n* = 9, P70 *n* = 12, P180 *n* = 9*; Fgfr3^Asn534Lys/+^*: P42 *n* = 8, P70 *n* = 10, P180 *n* = 10) was performed using an Instron 5543 load frame and 100 N load cell (Instron Limited). Bones were positioned horizontally on their dorsal surface and centered on supports with a span of 8 mm. Load was applied vertically to the mid-shaft with a constant rate of displacement of 2 mm/min until fracture and data were collected using BlueHill 3 software (Instron Limited).

L5 (*Fgfr3^+/+^:* P70 *n* = 12, *Fgfr3^Asn534Lys/+^:* P70 *n* = 10, P180 *n* = 10) vertebrae were processed using an HS Micro-Motor L50 (Henri Schein) with an H259 dentist drill (Komet Dental - Gebr. Brasseler GmbH & Co. KG) to erode all vertebral structures but the vertebral body (VB). L5 VBs were tested for compression using an Instron 5543 load frame and 500 N load cell (Instron Limited). To determine biomechanical variables for bone strength and toughness, the tested VB was placed standing on a support while the load was applied vertically to the top of the VB with a constant rate of displacement of 0.03 mm/s and data were acquired with BlueHill 3 software.

### Statistics.

Variables from all experiments were assessed for normality with Anderson-Daarling, Shapiro-Wilk, Kolmogorov, and D’Agostino-Pearson tests. Normally distributed data are expressed as mean ± SD. For analyses involving 2 groups, parametric 2-tailed Student’s *t* test was used. When 3 or more groups were analyzed, ANOVA with Bonferroni’s post hoc test was performed. When data were not normally distributed, Mann-Whitney test was used when 2 groups were analyzed (values are shown as mean ± SD).

Lc.Sph distribution, represented with violin plots, was determined by permutations of an equivalent number of lacunae from each sample. The volume and sphericity distribution for each mutant was compared to *Fgfr3^+/+^* using a 2-tailed Kolmogorov-Smirnov analysis (GraphPad Prism v8). Differences between mutant and *Fgfr3^+/+^* were considered valid if significant after 10 rounds of permutations.

The significance threshold was set at a *P* value of less than 0.05 (*P < 0.05, **P < 0.01, ***P < 0.001, ****P < 0.0001). Statistical analyses were performed using GraphPad Prism (v9).

### Study approval

All procedures were approved by the French Animal Care and Use Committee (APAFIS no. 26995).

## Author contributions

LL and DKE contributed equally to the work and should be considered co–first authors. LL and DKE designed and performed the experiments and LLM directed the research. AM, CV, ADLS, and NK performed some experiments. LL, DKE, AM, and YH analyzed the results and made the figures. AL and GB performed x-rays. DC, JHDB, and GRW participated in the project design. LL, DKE, and LLM wrote the manuscript. All authors reviewed and critically edited the manuscript.

## Supplementary Material

Supplemental data

## Figures and Tables

**Table 1:**
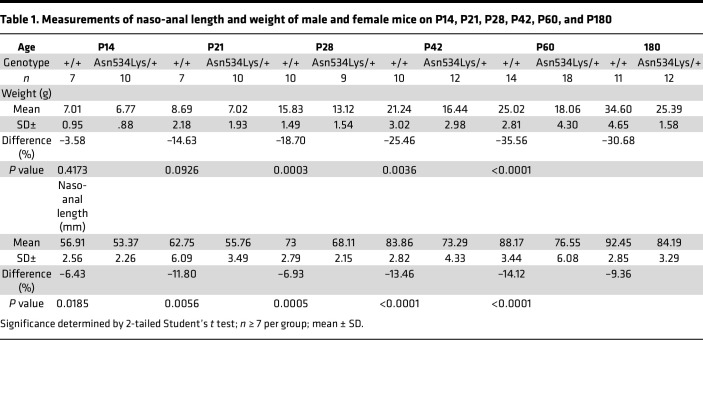
Measurements of naso-anal length and weight of male and female mice on P14, P21, P28, P42, P60, and P180

## Version 1 06/22/2023

Electronic publication
