# Supplementary material for: Hypochondroplasia gain-of-function mutation in FGFR3 causes defective bone mineralization in mice
Source: JCI Insight. 2023 Jun 22;8(12):e168796. doi: 10.1172/jci.insight.168796 (PMC10371252; doi:10.1172/jci.insight.168796)
Supplement: Supplemental data [file jciinsight-8-168796-s062.pdf]

Supplemental Figure 1

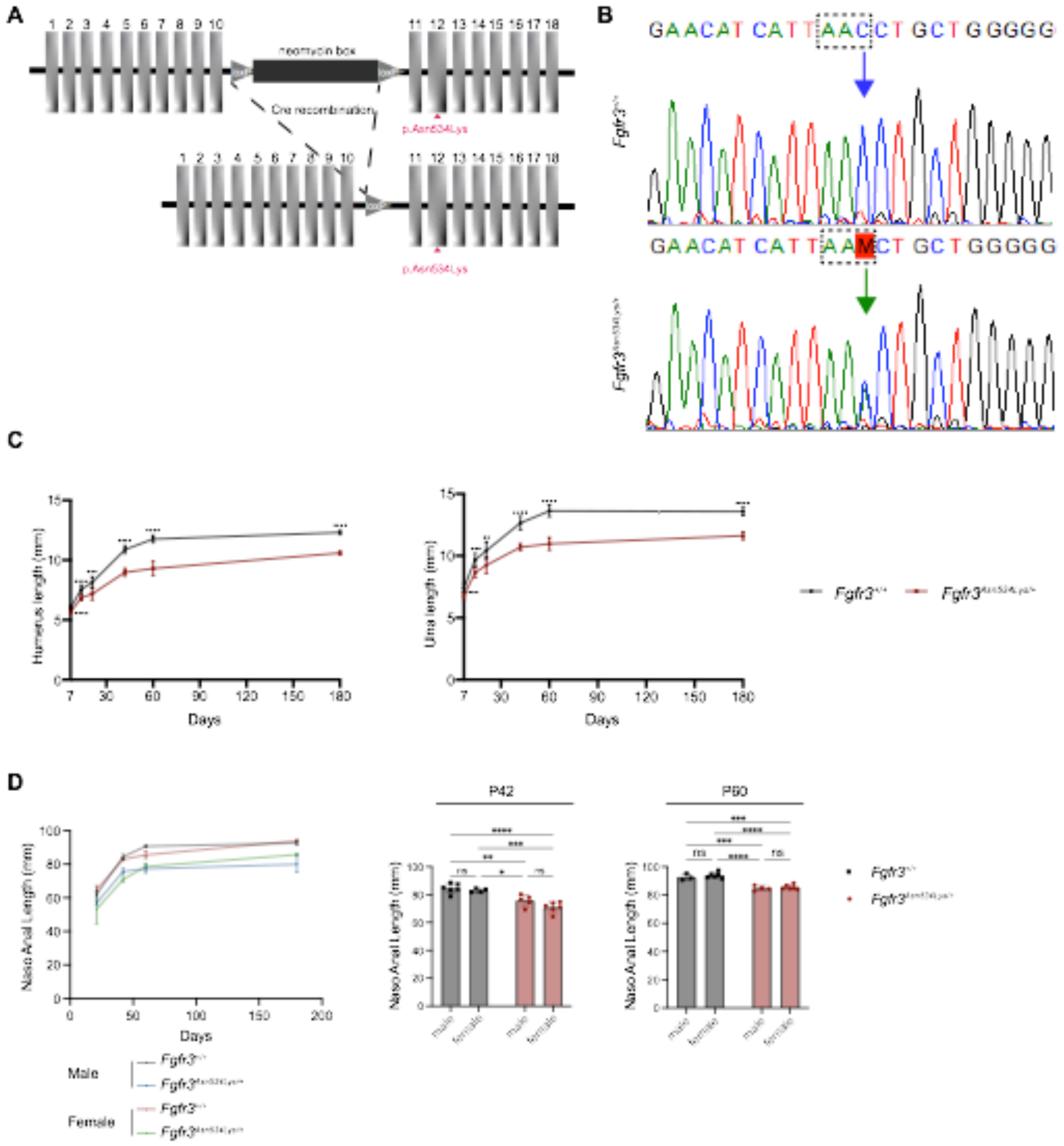

(**A**) Generation of the dwarf *Fgfr3*$^{Asn534Lys/+}$ mouse. The point mutation Asn534Lys is localized in mouse exon 12. *Fgfr3*$^{Asn534Lys/+}$ mice were crossed with CMV-Cre mice to remove the NEO cassette gene. (**B**) The presence of the heterozygous mutation p.Asn534Lys in DNA isolated from tail. (**C**) Graphical representation of the humerus and ulna length of *Fgfr3*$^{Asn534Lys/+}$ and *Fgfr3*$^{+/+}$ male and female mice during development (postnatal day 1, 7, 14, 21, 28, 42, 60 and 180) Student's t-test. (D) Graphical representation of the naso-anal length of *Fgfr3*$^{Asn534Lys/+}$ and *Fgfr3*$^{+/+}$ male and female mice during development (postnatal day 21,42, 60 and 180) (ns= not significant, * p<0.05, **p<0.01, ***p<0.001, ****p<0.0001 2-way ANOVA and Bonferroni's post-hoc test).

Supplemental Figure 2

A

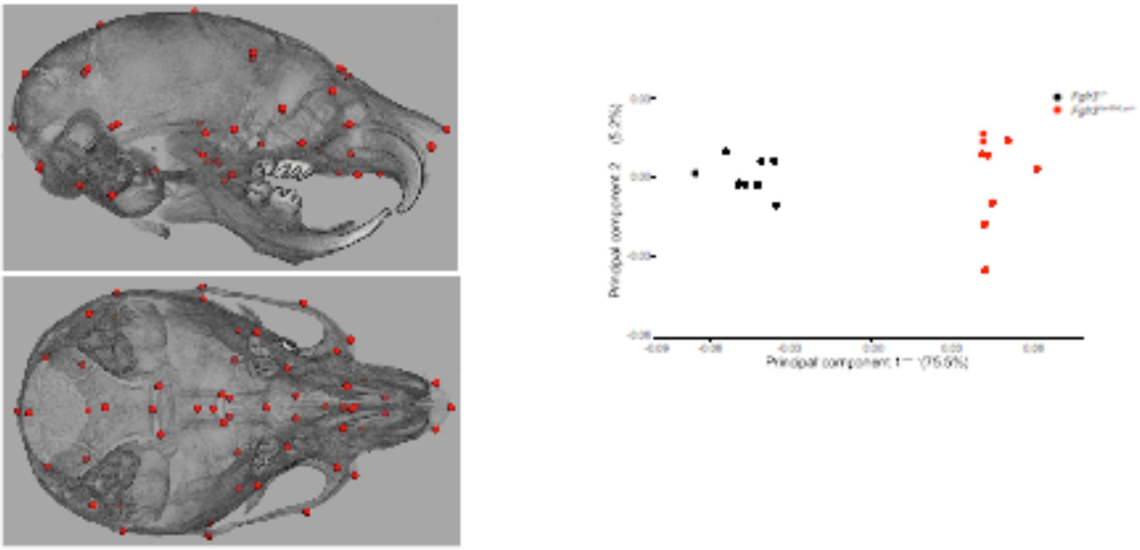

B

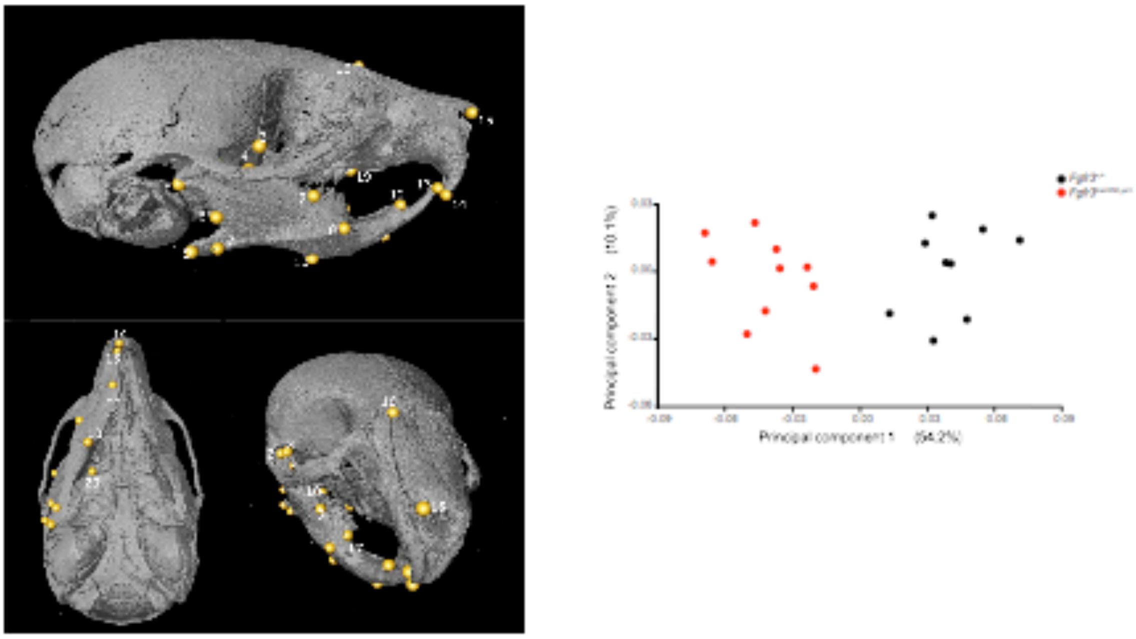

(**A**) Definitions and positioning of the 57 anatomical landmarks per specimen digitized on three-dimensional models on mouse skull. Comparison of skull shape between mutants and controls: plots of Principal Components (PC) scores for PC1 and PC2 with corresponding variances (in %) (*Fgfr3*$^{Asn534Lys/+}$ n=9 and *Fgfr3*$^{+/+}$ n=9). (**B**) Definitions and positioning of the 40 anatomical landmarks per specimen digitized on three-dimensional models: mandibular anatomical landmarks (n=20 per mandible); maxillar anatomical landmarks (n=10 per side). Comparison of mandibular shape and relationship to the maxilla between mutants and controls: plots of Principal Components (PC) scores for PC1 and PC2 with corresponding variances (in %) (*Fgfr3*$^{Asn534Lys/+}$ (n=9) and *Fgfr3*$^{+/+}$ (n=9). Results were statistically significant (p< 0.01) Student's t-test.

Supplemental Figure 3

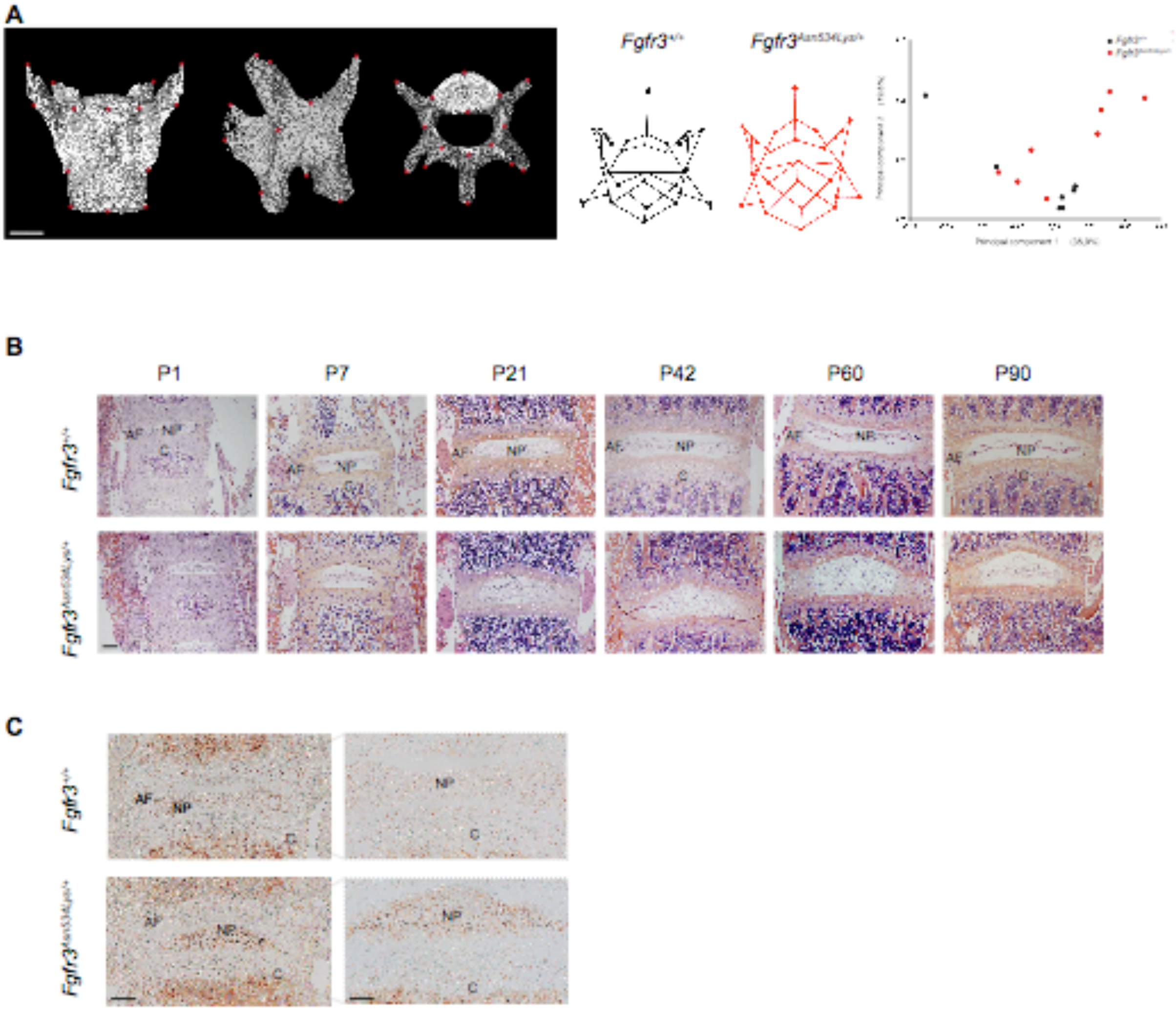

(**A)** Definitions and positioning of the 32 anatomical landmarks per specimen digitized on three-dimensional models on mouse L5 lumbar vertebrae. Comparison of L5 lumbar vertebrae shape between *Fgfr3*$^{+/+}$: P70 n=7, *Fgfr3*$^{Asn534Lys/+}$: P70 n=8 male mice plots of Principal Components (PC) scores for PC1 and PC2 with corresponding variances (in %). (**B**) H&E staining of L5 to L6 lumbar vertebrae intervertebral disc form 1 day-of-age to 3 months-of-age (scale bar=200 µm)**. (C)** Representative images of FGFR3 immunostaining (left panel: scale bar=200µm and right panel scale bar=100µm).

Supplemental Figure 4

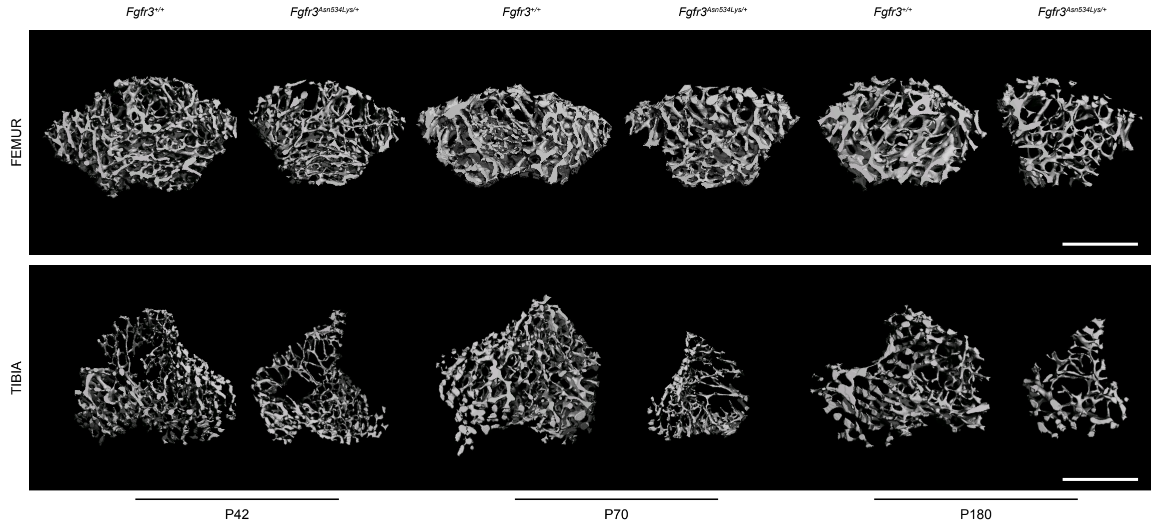

Representative µCT images of femur and tibia trabecular bone from *Fgfr3*$^{+/+}$ and *Fgfr3*$^{Asn534Lys/+}$ mice at P42, P70 and P180 (scale bars=1 mm).

Supplemental Figure 5

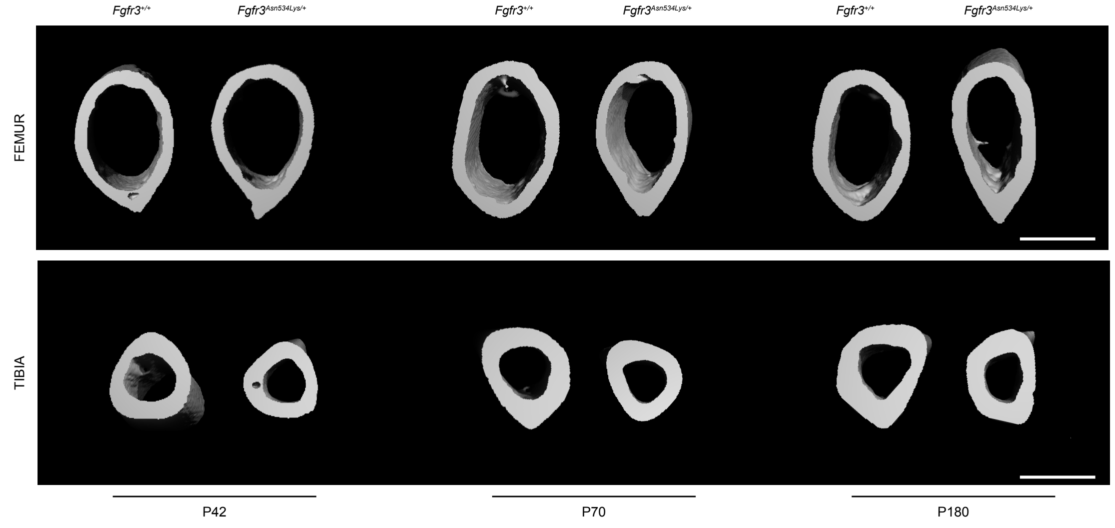

Representative µCT images of femur and tibia cortical bone from *Fgfr3*$^{+/+}$ and *Fgfr3*$^{Asn534Lys/+}$ mice at P42, P70 and P180 (scale bars=1 mm).

Supplemental Table 1

Age	P1		P7		P14		P21		P28		P42		P60		P180	
Genotype	+/+	*Asn534Lys/+*	+/+	*Asn534Lys/+*	+/+	*Asn534Lys/+*	+/+	*Asn534Lys/+*	+/+	*Asn534Lys/+*	+/+	*Asn534Lys/+*	+/+	*Asn534Lys/+*	+/+	*Asn534Lys/+*
n	10	13	7	11	7	10	7	10	10	9	10	12	14	18	11	12
**Weight (g)**																
Mean	1.36	1.38	3.41	3.65	7.01	6.77	8.69	7.02	15.83	13.12	21.24	16.44	25.02	18.06	34.60	25.39
SD ±	0.14	0.17	0.45	0.37	0.95	0.88	2.18	1.93	1.49	1.54	3.02	2.98	2.81	4.30	4.65	1.58
Difference (%)	1.99		6.66		-3.58		-14.63		-18.70		-25.46		-35.56		-30.68	
p value	0.7534		0.3623		0.4173		0.0926		0.0003		0.0036		<0.0001		<0.0001	
**Naso-anal length (mm)**																
Mean	31.22	30.54	41.69	39.76	56.91	53.37	62.75	55.76	73	68.11	83.86	73.29	88.17	76.55	92.45	84.19
SD ±	1.19	1.05	2.88	4.78	2.56	2.26	6.09	3.49	2.79	2.15	2.82	4.33	3.44	6.08	2.85	3.29
Difference (%)	-2.22		-4.75		-6.43		-11.80		-6.93		-13.46		-14.12		-9.36	
p value	0.1862		0.451		0.0185		0.0056		0.0005		<0.0001		<0.0001		<0.0001	

Age	P1		P7		P14		P21		P28		P42		P60		P180	
Genotype	+/+	*Asn534Lys/+*	+/+	*Asn534Lys/+*	+/+	*Asn534Lys/+*	+/+	*Asn534Lys/+*	+/+	*Asn534Lys/+*	+/+	*Asn534Lys/+*	+/+	*Asn534Lys/+*	+/+	*Asn534Lys/+*
n	0	0	7	12	8	12	9	11	0	0	10	12	6	8	11	11
**Femur length (mm)**																
Mean			6.10	5.58	8.36	7.38	9.37	8.02			12.99	10.58	14.90	11.20	15.52	12.58
SD ±			0.20	0.07	0.34	0.27	0.67	0.63			0.39	0.37	0.83	0.49	0.32	0.33
Difference (%)			-8.94		-12.41		-15.55				-20.47		-28.33		-20.93	
p value			0.000007		0.000006		0.000224				<0.000001		0.000002		<0.000001	
**Tibia length (mm)**																
Mean			7.43	6.85	11.22	9.56	12.37	10.37			16.03	12.54	17.53	13.14	18.06	14.02
SD ±			0.49	0.34	0.40	0.39	0.86	0.52			0.38	0.47	0.64	0.41	0.32	0.25
Difference (%)			-8.05		-15.90		-17.63				-24.46		-28.65		-25.20	
p value			0.0131		<0.0001		<0.0001				<0.0001		<0.0001		<0.0001	
**Humerus length (mm)**																
Mean			6.03	5.66	7.55	6.86	8.14	7.18			10.89	8.98	11.76	9.29	12.31	10.59
SD ±			0.16	0.20	0.26	0.36	0.53	0.45			0.32	0.31	0.61	0.30	0.15	0.17
Difference (%)			-6.20		-9.57		-12.53				-19.18		-23.41		-14.94	
p value			0.000063		0.00006		0.000414				<0.000001		<0.000001		<0.000001	
**Ulna length (mm)**																
Mean			7.41	6.78	9.67	8.65	10.42	9.24			12.65	10.69	13.61	10.97	13.57	11.62
SD ±			0.28	0.39	0.43	0.52	0.66	0.71			0.30	0.56	0.52	0.47	0.31	0.33
Difference (%)			-8.80		-11.17		-12.03				-16.79		-21.48		-15.50	
p value			0.000137		0.000133		0.001182				<0.000001		<0.000001		<0.000001	

Complete table of both male and female mice from P1 to P180. Naso-anal length, body weight, Femur length, Tibia length, Humerus length, Ulna length. Student's t-test, values presented as mean ± standard deviation.

Supplemental Table 2

Age	P14	
Genotype	+/+	*Asn534Lys/+*
n	9	8
**Skull length (mm)**		
mean	17.88	16.3
SD ±	0.6756	0.4993
difference (%)	8.84	
p value	<0.0001	
**Skull width (mm)**		
mean	8.862	9.21
SD ±	0.1869	0.2692
difference (%)	-3.93	
p value	0.007	
**Centroid size**		
mean	41.98	39.74
SD ±	0.9691	0.69
difference (%)	5.34	
p value	<0.0001	
**Skull base length (mm)**		
mean	7.596	6.008
SD ±	0.2319	0.129
difference (%)	20.91	
p value	0.02	
**Foramen magnum area (mm^2)**		
mean	13.07	10.59
SD ±	0.6024	0.7466
difference (%)	18.97	
p value	<0.0001	

Skull analyses on P14 mice, Student’s t-test.

Supplemental Table 3

			P42			P70			P180		
**Analysis**	**Bone**	**Genotype**	**Bone %**	**Min size (µm)**	**Max size (µm)**	**Bone %**	**Min size (µm)**	**Max size (µm)**	**Bone %**	**Min size (µm)**	**Max size (µm)**
**Trabecular**	Lumbar Vertebra 5	+/+				100%*	3260	3460			
		Asn534Lys/+					2605	3165			
	Femur	+/+	7.0%	955	1040	6.0%	935	955	6.0%	905	975
		Asn534Lys/+		765	855		685	795		765	840
	Tibia	+/+	6.0%	945	1040	6.0%	1070	1090	5.5%	975	1040
		Asn534Lys/+		700	825		780	845		740	820
**Cortical**	Femur	+/+	9.5%	1290	1410	8.5%	1320	1350	8.0%	1210	1300
		Asn534Lys/+		980	1160		970	1130		1030	1120
	Tibia	+/+	9.5%	1500	1640	8.5%	1510	1540	8.0%	1420	1510
		Asn534Lys/+		1110	1310		1100	1200		1080	1190

** For Lumbar Vertebrae the full Vertebral Body (in between the two cartilaginous end plates) was selected as ROI*

Selection of region of interests (ROIs) for µCT analyses of lumbar vertebrae, femurs and tibiae. ROIs expressed as % of total bone length, minimum and maximum size per group.

Supplemental Table 4

		Genotype	P70 (+/+ **n**=11; *Asn534Lys/+* **n**=10) Mean	SD ±	Difference (%)	p value
Trabecular	TV (mm^3)	+/+	2.893	0.1775	-26.24	<0.0001
		Asn534Lys/+	2.134	0.4106		
	BV/TV	+/+	0.3508	0.02244	-17.65	<0.0001
		Asn534Lys/+	0.2889	0.03187		
	Tb.N (mm^{-1})	+/+	6.527	0.175	-0.54	0.7922
		Asn534Lys/+	6.492	0.4171		
	Tb.Th (mm)	+/+	0.06207	0.003405	-14.63	<0.0001
		Asn534Lys/+	0.05299	0.0031		
	Tb.Sp (mm)	+/+	0.1576	0.005307	-0.25	0.8933
		Asn534Lys/+	0.1572	0.01042		
	BMD ($mgHAcm^{-3}$)	+/+	646.1	16.84	-6.53	<0.0001
		Asn534Lys/+	603.9	12.75		
	Vertebral body size (mm)	+/+	3.338	0.05025	-11.89	<0.0001
		Asn534Lys/+	2.941	0.1842		

Lumbar vertebrae 5 measurements obtained with µCT are presented classified by genotype and age. Values are expressed as mean ± standard deviation. The difference among *Fgfr3*$^{+/+}$ and *Fgfr3*$^{Asn534Lys/+}$ mice is also presented in percentage and the relative p-value is also reported.

Supplemental Table 5

			P42				P70				P180			
			+/+ **n**=9; *Asn534Lys/+* **n**=8				+/+ **n**=12; *Asn534Lys/+* **n**=10				+/+ **n**=9; *Asn534Lys/+* **n**=10			
		Genotype	Mean	SD ±	Difference (%)	p value	Mean	SD ±	Difference (%)	p value	Mean	SD ±	Difference (%)	p value
Trabecular	TV (mm^3)	+/+	2.196	0.1748	-20.67	<0.0001	2.338	0.1166	-24.85	<0.0001	2.441	0.1993	-28.10	<0.0001
		Asn534Lys/+	1.742	0.3111			1.757	0.2102			1.755	0.1118		
	BV/TV	+/+	0.197	0.03725	-29.70	0.0038	0.231	0.02938	-40.48	<0.0001	0.1916	0.03649	-37.37	0.0002
		Asn534Lys/+	0.1385	0.02993			0.1375	0.04843			0.12	0.02594		
	Tb.N (mm^{-1})	+/+	5.713	0.3253	-14.32	0.0007	5.843	0.2331	-16.70	<0.0001	4.418	0.3919	-9.41	0.118
		Asn534Lys/+	4.895	0.4552			4.867	0.7461			4.002	0.2078		
	Tb.Th (mm)	+/+	0.0462	0.005121	-6.52	0.4208	0.05291	0.004729	-16.95	<0.0001	0.05718	0.00389	-12.89	0.0009
		Asn534Lys/+	0.04319	0.004262			0.04394	0.003564			0.04981	0.002846		
	Tb.Sp (mm)	+/+	0.1656	0.01105	18.60	0.0136	0.1544	0.00697	28.30	<0.0001	0.2085	0.02071	12.33	0.0342
		Asn534Lys/+	0.1964	0.02539			0.1981	0.03735			0.2342	0.01347		
	BMD ($mgHAcm^{-3}$)	+/+	716.4	23.37	1.10	0.787	785.8	17.55	-3.96	0.0013	837	17.76	-2.51	0.0658
		Asn534Lys/+	724.3	24.79			754.7	19.69			816	12.72		

Femur measurements obtained with µCT are presented classified by genotype and age. Values are expressed as mean ± standard deviation. The difference among *Fgfr3*$^{+/+}$ and *Fgfr3*$^{Asn534Lys/+}$ mice is also presented in percentage and the relative p-value is also reported.

Supplemental Table 6

			P42				P70				P180			
			+/+ n=9; *Asn534Lys/+* n=8				+/+ n=12; *Asn534Lys/+* n=10				+/+ n=9; *Asn534Lys/+* n=10			
		**Genotype**	**Mean**	**SD ±**	**Difference (%)**	**p value**	**Mean**	**SD ±**	**Difference (%)**	**p-value**	**Mean**	**SD ±**	**Difference (%)**	**p value**
Trabecular	TV (mm^3)	+/+	2.301	0.3118	-46.07	<0.0001	2.736	0.202	-46.56	<0.0001	2.176	0.4041	-49.08	<0.0001
		Asn534Lys/+	1.241	0.2278			1.462	0.3008			1.108	0.0652		
	BV/TV	+/+	0.1184	0.02636	-16.27	0.7526	0.1987	0.02861	-38.35	<0.0001	0.1883	0.03787	-24.00	0.0198
		Asn534Lys/+	0.09914	0.01818			0.1225	0.05145			0.1431	0.03077		
	Tb.N (mm^{-1})	+/+	4.641	0.6775	-5.24	>0.9999	5.852	0.217	-18.11	0.0003	4.542	0.4489	-4.80	>0.9999
		Asn534Lys/+	4.398	0.477			4.792	1.035			4.324	0.3388		
	Tb.Th (mm)	+/+	0.0379	0.003189	-3.67	>0.9999	0.04822	0.003218	-15.45	<0.0001	0.05625	0.002492	-7.04	0.0354
		Asn534Lys/+	0.03651	0.004308			0.04077	0.003519			0.05229	0.003301		
	Tb.Sp (mm)	+/+	0.2094	0.03547	7.26	0.9389	0.1504	0.007008	36.30	0.0004	0.2015	0.02273	7.64	0.8387
		Asn534Lys/+	0.2246	0.03404			0.205	0.05082			0.2169	0.02043		
	BMD ($mgHAcm^{-3}$)	+/+	694.9	18.81	0.78	0.9333	785.2	15.02	-4.30	0.0012	856.3	11.54	-1.13	0.4941
		Asn534Lys/+	700.3	27.67			751.4	27.19			846.6	23.06		

Tibia measurements obtained with μCT are presented classified by genotype and age. Values are expressed as mean ± standard deviation. The difference among *Fgfr3*$^{+/+}$ and *Fgfr3*$^{Asn534Lys/+}$ mice is also presented in percentage and the relative p-value is also reported. (2-way ANOVA with Bonferroni's test).

Supplemental Table 7

			P42				P70				P180			
			+/+ n=9; *Asn534Lys/+* n=8				+/+ n=12; *Asn534Lys/+* n=10				+/+ n=9; *Asn534Lys/+* n=10			
		**Genotype**	**Mean**	**SD ±**	**Difference (%)**	**p-value**	**Mean**	**SD ±**	**Difference (%)**	**p-value**	**Mean**	**SD ±**	**Difference (%)**	**p-value**
Tibia - Fracture	Yield Load (N)	+/+	7.36	1.72	-34.91	0.0049	10.11	1.637	-38.12	<0.0001	13.1	1.885	-17.63	0.0077
		Asn534Lys/+	4.79	1.36			6.256	0.9319			10.79	1.791		
	Maximum Load (N)	+/+	9.96	1.64	-24.07	0.0126	14	1.306	-37.14	<0.0001	19.75	1.427	-25.57	<0.0001
		Asn534Lys/+	7.57	2.32			8.801	1.563			14.7	1.669		
	Fracture Load (N)	+/+	6.65	2.58	-29.78	0.3462	8.88	3.228	-29.13	0.0642	12.67	2.529	1.50	>0.9999
		Asn534Lys/+	4.67	2.19			6.293	1.911			12.86	2.402		
	Stiffness (Nmm^{-1})	+/+	38.75	8.11	-37.01	0.0014	53.95	7.539	-52.99	<0.0001	97.01	9.889	-42.46	<0.0001
		Asn534Lys/+	24.41	8.85			25.36	7.662			55.82	5.003		
	Energy Dissipated at Fracture (%)	+/+	88.85	13.45	-4.16	>0.9999	81.93	19.76	-4.87	>0.9999	86.71	12.38	-42.58	0.0001
		Asn534Lys/+	85.15	19.49			77.94	17			49.79	23.05		
	Plastic Work / Total Work (%)	+/+	88.96	7.20	-0.02	>0.9999	83.31	7.502	-3.34	>0.9999	87.31	6.726	-23.90	<0.0001
		Asn534Lys/+	88.94	7.81			80.53	6.836			66.44	9.939		
L5 - Compression	Yield Load (N)	+/+					18.72	7.493	-20.94	0.1531				
		Asn534Lys/+					14.8	3.979						
	Maximum Load (N)	+/+					26.51	5.768	-16.75	0.0481				
		Asn534Lys/+					22.07	3.667						
	Stiffness (Nmm-1)	+/+					148.5	41.01	-34.01	0.0044				
		Asn534Lys/+					97.99	30.92						

Biomechanical test data. The table presents the data of our studies sorted per genotype and age. They are expressed as mean ± standard deviation. The variations across *Fgfr3*$^{+/+}$ and *Fgfr3*$^{Asn534Lys/+}$ mice are expressed in percentage and their p-values are listed. Tibia 3-point bending test (2-way ANOVA with Bonferroni's test) and L5 Vertebra compression (Unpaired Student's t test).

Supplemental Table 8

Gene/Accession number	Gene name (Mus Musculus)	Gene Function	5’- Forward primer – 3’ length/Tm/%GC/Self Comp/Self-3’-Comp	5’- Reverse primer – 3’ length/Tm/%GC/Self Comp/Self-3’-Comp	Amplicon (length, efficiency, R^2)
Runx2/ NM_001145920	Runt-related transcription factor 2	Osteoblast differentiation	5′-TGGCCACTTACCACAGAGCTATT-3′ 23bp/61.65°C/47.83%/6/2	5′-AGGTTTAGAGTCATCAAGCTTCTGTC-3′ 26bp/60.80°C/42.31%/6/4	89bp, 100.63%, 0.99
Col1a1/ NM_007742	Type I collagen	Bone formation	5′-CTCAGAGGCGAAGGCAACA-3′ 19bp/60.00°C/57.89%/5/0	5′-TGACTGTCTTGCCCCAAGTTC-3′ 21bp/60.48°C/52.38/%/5/1	88bp, 98.19%, 0.9973
Ocn (Bglap3)/ NM_031368	Osteocalcin	Bone formation	5′-TGGCTGCGCTCTGTCTCT-3′ 18bp/60.99°C/61.11%/4/0	5′- TTATTGCCCTCCTGCTTGG-3′ 19bp/57.41°C/52.63%/2/0	91bp, 86.80%, 0.99
Trap (Acp5)/ NM_001102405.1	Tartrate-resistant acid phosphatase (TRAP)	Bone resorption	5′-CGACCATTGTTAGCCACATACG-3’ 22bp/59.72°C/50.00%/4/2	5′-ACACCGTTCTCGTCCTGAAG-3′ 20bp/59.69°C/55.00%/3/2	86bp, 94.96%, 0.99
Gapdh/ NM_001289726.2	Glyceraldehyde 3-phosphate dehydrogenase	Cell metabolism (Housekeeping gene)	5′-GGGCTGGCATTGCTCTCA-3’ 18bp/60.04°C/61.11%/5/1	5′- GGTCCACCACCCTGTTGCT-3′ 19bp/62.13°C/63.16%/1/2	87bp, 99.25%, 0.95
Osx (Sp7)/ NM_130458	Transcription factor Sp7 (Osterix)	Osteoblast differentiation regulator	5′-CGCTTTGTGCCTTTGAAAT-3′ 19bp/55.20°C/42.11%/5/3	5′-CCGTCAACGACGTTATGC-3’ 18bp/56.68°C/55.56%/7/3	127bp, 114.35%, 0.99
Dkk1/ NM_010051.3	Dickkopf-related protein 1	Bone formation inhibitor	5'-TCCCAGAAGAACCACACTGACTTC-3' 24bp/62.26°C/50.00%/4/2	5'- TCTTGGACCAGAAGTCTCTTGCAC-3' 24bp/62.54°C/50.00%/5/2	122bp, 120.92%, 0.89
Sost/ NM_024449	Sclerostin	Bone formation inhibitor	5'- ATCCCAGGGCTTGGAGAGTA-3' 20bp/59.66°C/55.00%/6/2	5'-ACATCTTTGGCGTCATAGGG-3' 20bp/57.67°C/50.00%/6/2	110bp, 102.08%, 0.97

Primer sequences and amplification characteristics of the targeted genes.

Uncropped gel P-ERK1/2 (10 min exposition)

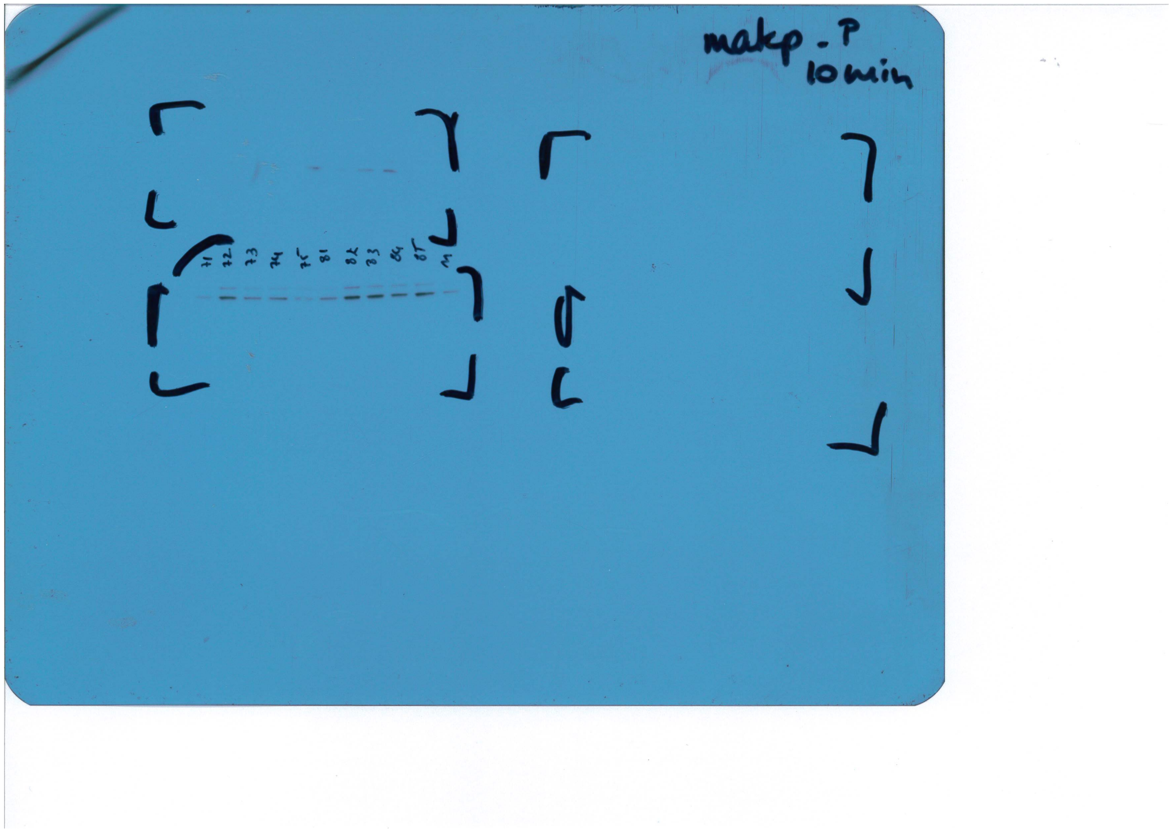

Samples number :
71: *Fgfr3*$^{+/+}$ FGF2 0 min
72 : *Fgfr3*$^{+/+}$ FGF2 5 min
73 : *Fgfr3*$^{+/+}$ FGF2 30 min
74 : *Fgfr3*$^{+/+}$ FGF2 60 min
75 : *Fgfr3*$^{+/+}$ FGF2 120 min
81 : *Fgfr3*$^{+/Asn534Lys}$ FGF2 0 min
82 : *Fgfr3*$^{+/Asn534Lys}$ FGF2 5 min
83 : *Fgfr3*$^{+/Asn534Lys}$ FGF2 30 min
84 : *Fgfr3*$^{+/Asn534Lys}$ FGF2 60 min
85 : *Fgfr3*$^{+/Asn534Lys}$ FGF2 120 min
11: internal control in the gel

Uncropped gel : ERK1/2 (30 min exposition)

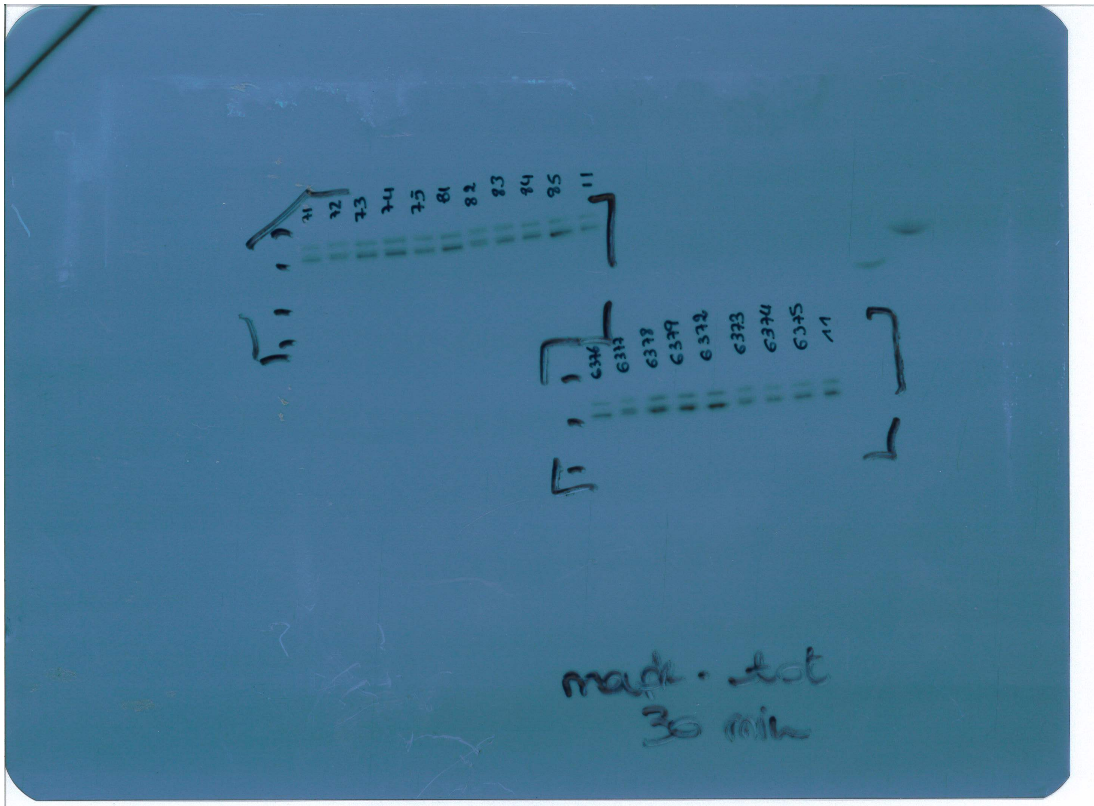

Samples number :

71: *Fgfr3*$^{+/+}$ FGF2 0 min

72 : *Fgfr3*$^{+/+}$ FGF2 5 min

73 : *Fgfr3*$^{+/+}$ FGF2 30 min

74 : *Fgfr3*$^{+/+}$ FGF2 60 min

75 : *Fgfr3*$^{+/+}$ FGF2 120 min

81 : *Fgfr3*$^{+/Asn534Lys}$ FGF2 0 min

82 : *Fgfr3*$^{+/Asn534Lys}$ FGF2 5 min

83 : *Fgfr3*$^{+/Asn534Lys}$ FGF2 30 min

84 : *Fgfr3*$^{+/Asn534Lys}$ FGF2 60 min

85 : *Fgfr3*$^{+/Asn534Lys}$ FGF2 120 min

11: internal control in the gel

Uncropped gel : actin (30 sec exposition)

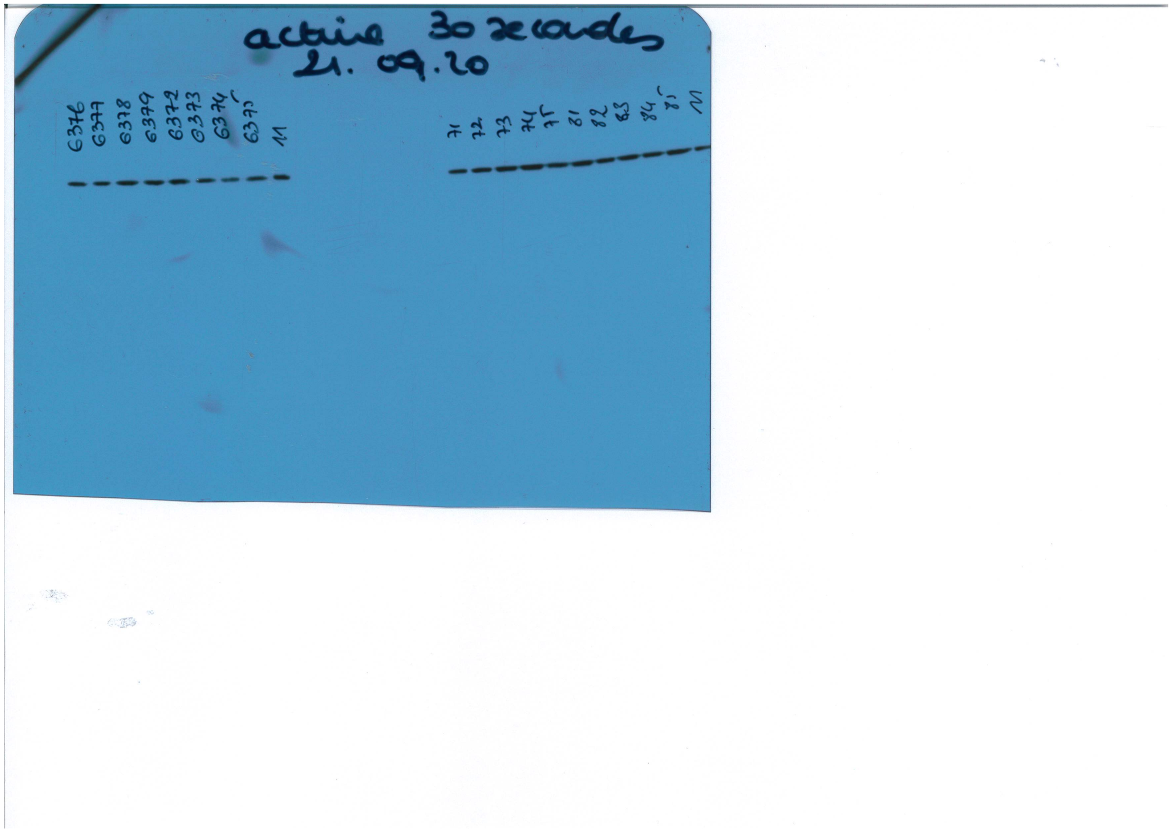

Samples number :

71: *Fgfr3*$^{+/+}$ FGF2 0 min

72 : *Fgfr3*$^{+/+}$ FGF2 5 min

73 : *Fgfr3*$^{+/+}$ FGF2 30 min

74 : *Fgfr3*$^{+/+}$ FGF2 60 min

75 : *Fgfr3*$^{+/+}$ FGF2 120 min

81 : *Fgfr3*$^{+/Asn534Lys}$ FGF2 0 min

82 : *Fgfr3*$^{+/Asn534Lys}$ FGF2 5 min

83 : *Fgfr3*$^{+/Asn534Lys}$ FGF2 30 min

84 : *Fgfr3*$^{+/Asn534Lys}$ FGF2 60 min

85 : *Fgfr3*$^{+/Asn534Lys}$ FGF2 120 min

11: internal control in the gel
